# Halogen-bonding-induced diverse aggregation of 4,5-diiodo-1,2,3-triazolium salts with different anions

**DOI:** 10.3762/bjoc.16.10

**Published:** 2020-01-13

**Authors:** Xingyu Xu, Shiqing Huang, Zengyu Zhang, Lei Cao, Xiaoyu Yan

**Affiliations:** 1 Department of Chemistry, Renmin University of China, Beijing 100872, People’s Republic of China

**Keywords:** aggregation, 4,5-diiodo-1,2,3-triazolium salts, halogen bond, non-covalent interaction

## Abstract

The synthesis of 4,5-diiodo-1,3-dimesityl-1,2,3-triazolium salts with different anions have been developed. These triazolium salts show diverse aggregation via halogen bonding between C–I bonds and anions. Triazolium with halide anions exists as a tetramer with saddle conformation. Triazolium tetrafluoroborate exists as a trimer with Chinese lantern shape conformation. Triazolium trifluoroacetate and acetate exist as dimers, respectively, while the former shows boat conformation and the latter forms rectangle conformation. Triazolium salts form a linear polymer with polyiodide.

## Introduction

**Figure 1:**
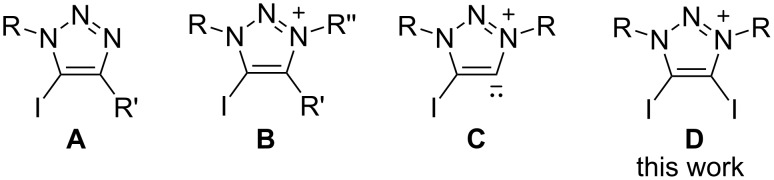
1,2,3-Triazole based XB donors: 1,2,3-triazole **A**, 1,2,3-triazolium **B**, 1,2,3-triazolylidene **C** and diiodotriazolium **D**.

The halogen bond (XB) is a noncovalent interaction between electrophilic halides and Lewis bases or electron-rich regions [1–2]. Computational studies [3–7] and cyrstal architectures including XB-donors (σ-hole) such as perfluorocarbons [8–12], tetraiodoethylene [13], 1,2-diiodo-1,2-difluoroethene [14], diiodoacetylene [15] and iodo/bromoethynyl moieties [16] have revealed that the XB-donors interacting with XB-acceptors (a nucleophilic region) are in approximately linear orientation. Besides, linearity, tunability and hydrophobicity (features of the XB ) are widely applied in crystal engineering, supramolecular chemistry, anion recognition, organocatalysis, materials science and tuning of biomolecular systems [17–27]. 1,2,3-Triazole-based XB-donors, such as 5-iodo-1,2,3-triazoles **A** [28–33] and 5-iodo-1,2,3-triazolium **B** [34–37] (Figure 1), are promising candidates for XB donors, which is mainly due to the ease of preparation via a copper-catalyzed click reaction between azide and alkyne [38–39]. 1,2,3-Triazoles and 1,2,3-triazolium-based XB activators have been found applications in catalytic reactions [40–41] and anion recognition [42]. Recently, we reported neutral 4-halo-1,2,3-triazolylidenes **C** [43], which had a carbene character with σ-donation at the carbon and a σ-hole at the halogen atom. XB is observed by single-crystal X-ray diffraction in their coinage metal complexes. Meanwhile, 4-bromo-1,2,3-triazolylidene can catalyze H/D exchange of aldehydes [44]. Despite a variety of XB donors based on 1,2,3-triazole have been reported, no 4,5-diido-1,2,3-triazolium salts have been reported for an XB interaction. Herein, we report the synthesis and characterization of 4,5-diido-1,2,3-triazolium **D** with different anions. The crystal structures of these compounds show XB interactions between the triazolium moiety and anions, and different aggregations are formed. Triazolium with halide anions exists as tetramers with saddle conformation. Triazolium tetrafluoroborate exists as trimer with Chinese lantern shape conformation. Triazolium trifluoroacetate and acetate exist as dimers, respectively, while the former shows a boat conformation and the latter forms a rectangle conformation. Triazolium salts form a linear polymer with polyiodide.

## Results and Discussion

**Scheme 1:**
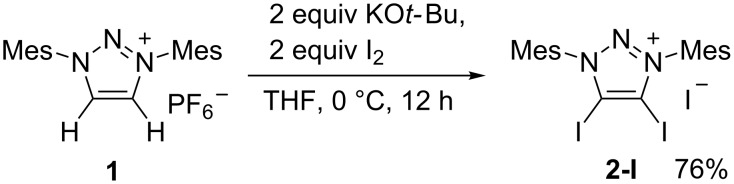
Synthesis of 4,5-diiodo-1,3-dimesityl-1,2,3-triazolium with iodide, Mes: 2,4,6-Me_3_C_6_H_2_.

Recently, we found that 4-iodotriazolylidene can be prepared by the treatment of a 4,5-unsubstituted triazolium salt with one equivalent I_2_ in the presence of two equivalents of potassium *tert*-butoxide [43]. When 4,5-unsubstituted triazolium salt **1** was treated with two equivalents I_2_ and two equivalents of potassium *tert*-butoxide, 4,5-diiodo-1,3-dimesityl-1,2,3-triazolium **2-I** was synthesized in a good yield (Scheme 1). The product **2-I** was characterized by ^1^H NMR, ^13^C NMR, and high-resolution mass spectrometry. **2-I** has a poor solubility in most organic solvents such as dichloromethane, trichloromethane, tetrahydrofuran, and ethanol. A single crystal of **2-I** was obtained by slow diffusion of ether into dimethylformamide solution.

**Scheme 2:**
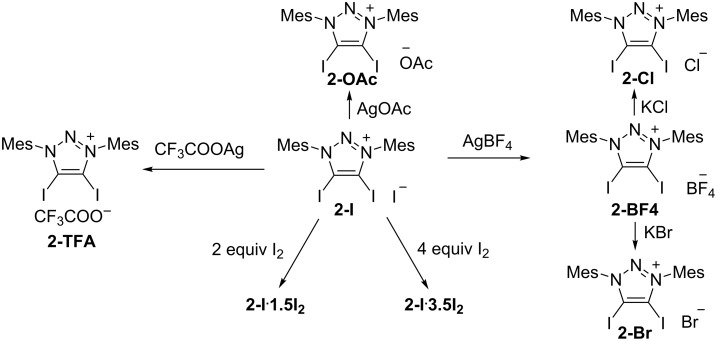
Synthesis of 4,5-diiodo-1,3-dimesityl-1,2,3-triazolium with different anion.

Ion exchange of **2-I** with AgBF_4_ afforded **2-BF_4_**. In contrast, **2-BF_4_** was soluble in dichloromethane and trichloromethane. A single crystal of **2-BF_4_** was obtained by slow diffusion of ether into a dichloromethane solution. In a similar manner, **2-OAc** and **2-TFA** were obtained via removing the iodide anion by AgOAc and CF_3_COOAg. A single crystal of **2-OAc** suitable for X-ray diffraction analysis was obtained by slow diffusion of *n*-pentane into a dichloromethane solution. A single crystal of **2-TFA** suitable for X-ray diffraction analysis was obtained by slow evaporation of dichloromethane solution (Scheme 2).

**2-Br** and **2-Cl** were synthesized by ion exchange between **2-BF_4_** and the respective potassium halide in acetonitrile. A single crystal of **2-Br** was obtained by slow diffusion of *n*-pentane into a dichloromethane solution. While the single crystal of **2-Cl** was obtained by slow evaporation of dichloromethane solution. The treatment of **2-I** with 2 equivalents iodine or 4 equivalents iodine afforded triazolium polyiodide **2-I^.^1.5I_2_** and **2-I^.^3.5I_2_**, respectively. The single crystals were obtained by slow diffusion of ether into a dichloromethane solution.

**Figure 2:**
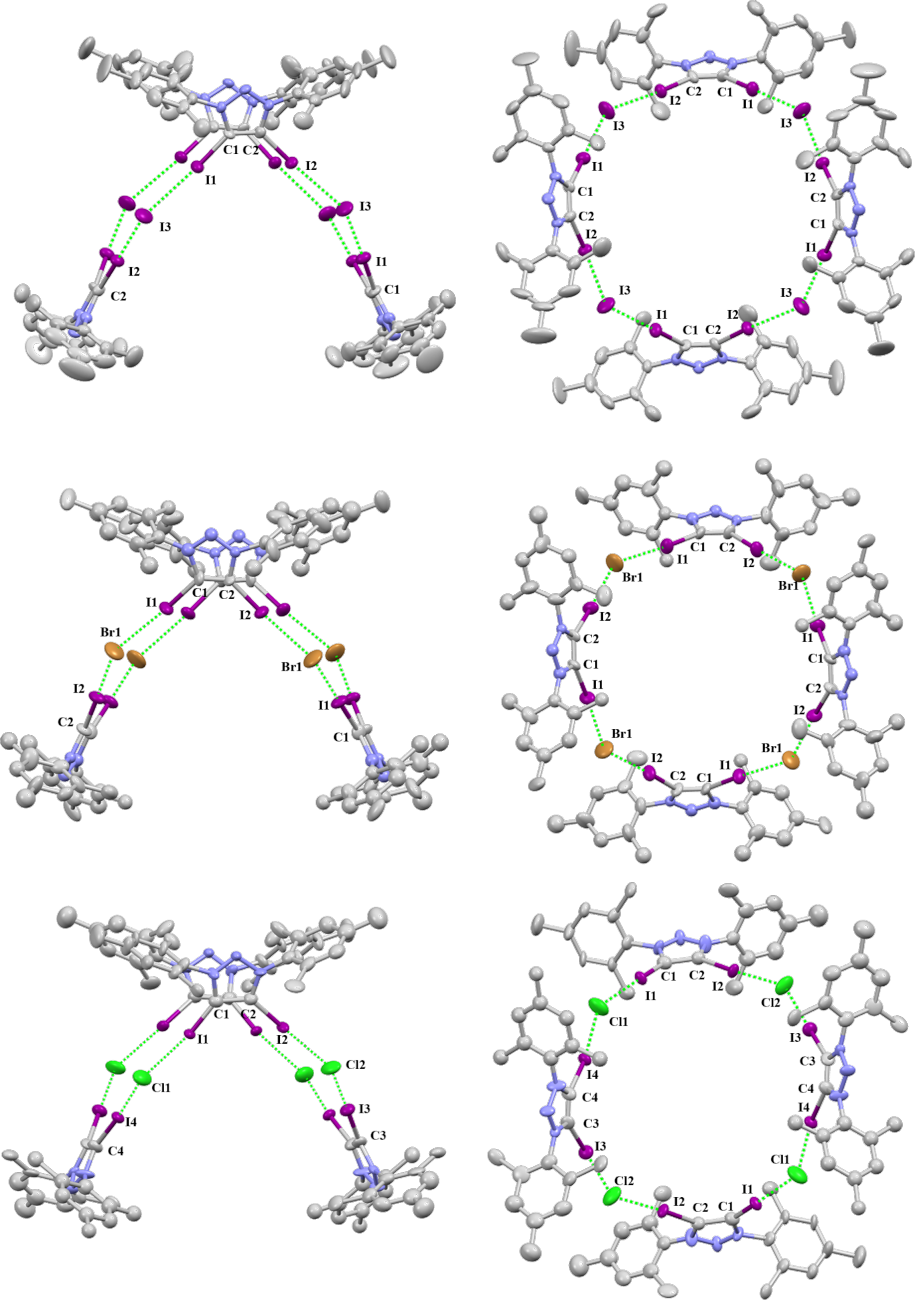
Packing structure of **2-I** (top), **2-Br** (middle) and **2-Cl** (bottom). Hydrogen atoms have been omitted for clarity. Side view (left) and top view (right).

The crystal X-ray analyses of **2-I, 2-Br** and **2-Cl** show tetrameric aggregation of the 4,5-diiodo-1,3-dimesityl-1,2,3-triazolium moiety with four anion halides that are bridged together to form a saddle shape through XB (Figure 2). **2-I** crystallizes in the tetragonal space group 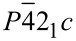. The distances of I···I are 3.306(1) Å and 3.300(1) Å which are due to the XB. The C–I···I angles are 176.9(3)° and 176.2(3)° (Table 1). The I···I distance is short: the reduction ratio R_XB_ [45], defined as the ratio of the actual distance over the sum of van der Waals radii, amounts to 0.81. The **2-Br** crystal package has a similar package diagram. **2-Br** crystallizes in the tetragonal space group 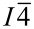. The distances of I···Br are 3.107(2) Å, 3.123(2) Å, the C–I···Br angles are 177.1(4)°, 173.4(6)°, and the R_XB_ values are 0.80. The crystal **2-Cl** has a monoclinic crystal system and the space group is *C*2. The distances of I···Cl are 2.963(9) Å, 2.989(6) Å, 2.934(7) Å and 2.98(1) Å. The C–I···Cl angles are 176.8(6)°, 173.4(6)°, 176.2(6)° and 175.9(6)°. The R_XB_ values are 0.77. These crystal package diagrams display that a bent arrangement of the XB donors are around the central halide anion. The measured bent angles of **2-I**, **2-Br** and **2-Cl** by mercury [46] are 146.38(3)°, 144.12(7)° and 145.6(3)°.

**Figure 3:**
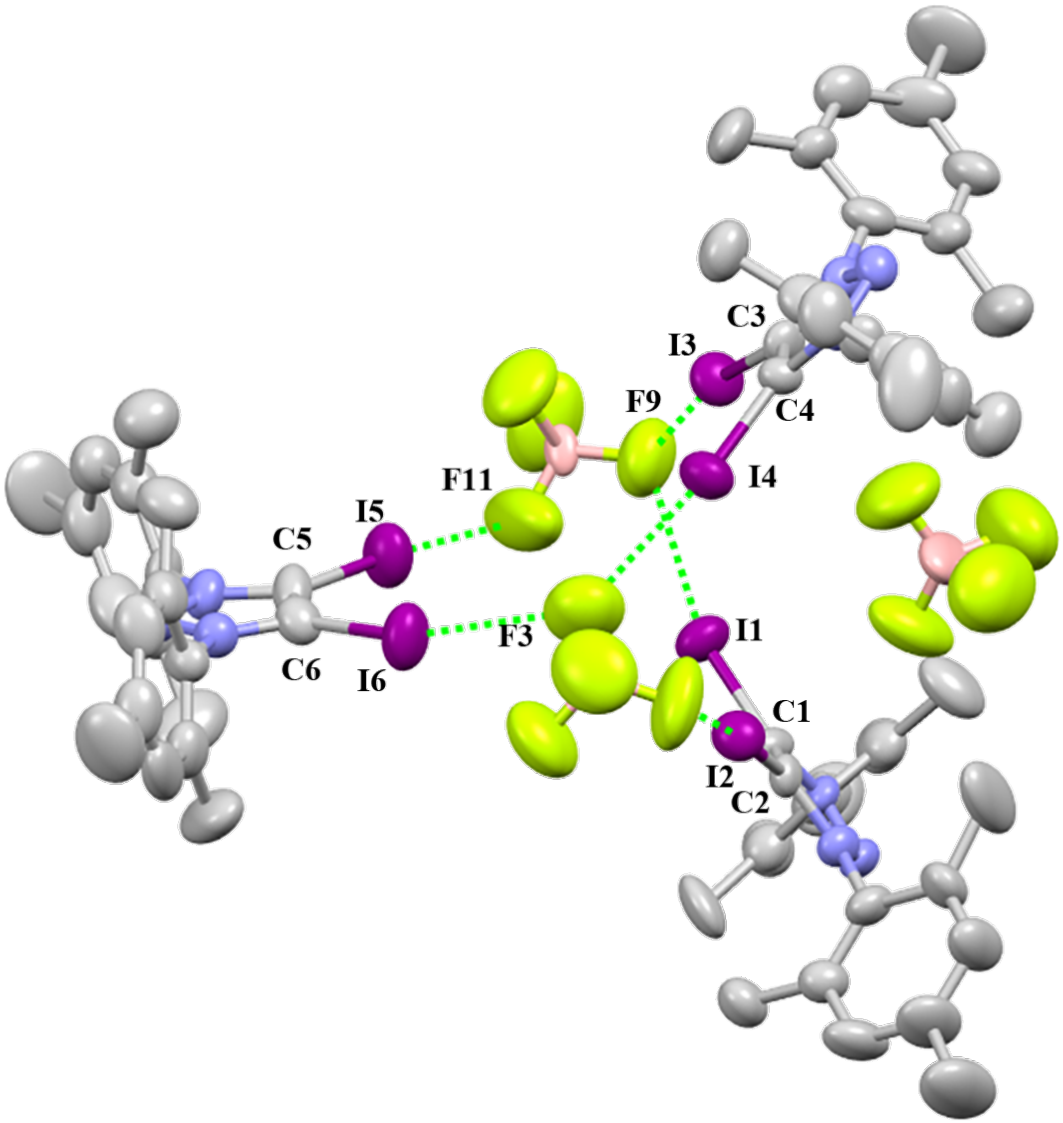
Packing structure of **2-BF_4_**. Hydrogen atoms have been omitted for clarity.

The crystal X-ray analyses of **2-BF_4_** shows that the diiodotriazolium moiety has formed with the tetrafluoroborate anion a triangle in which only two anions and three cations are assembled and one tetrafluoroborate is independent (Figure 3). **2-BF_4_** crystallizes in the triclinic space group 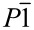.

**Figure 4:**
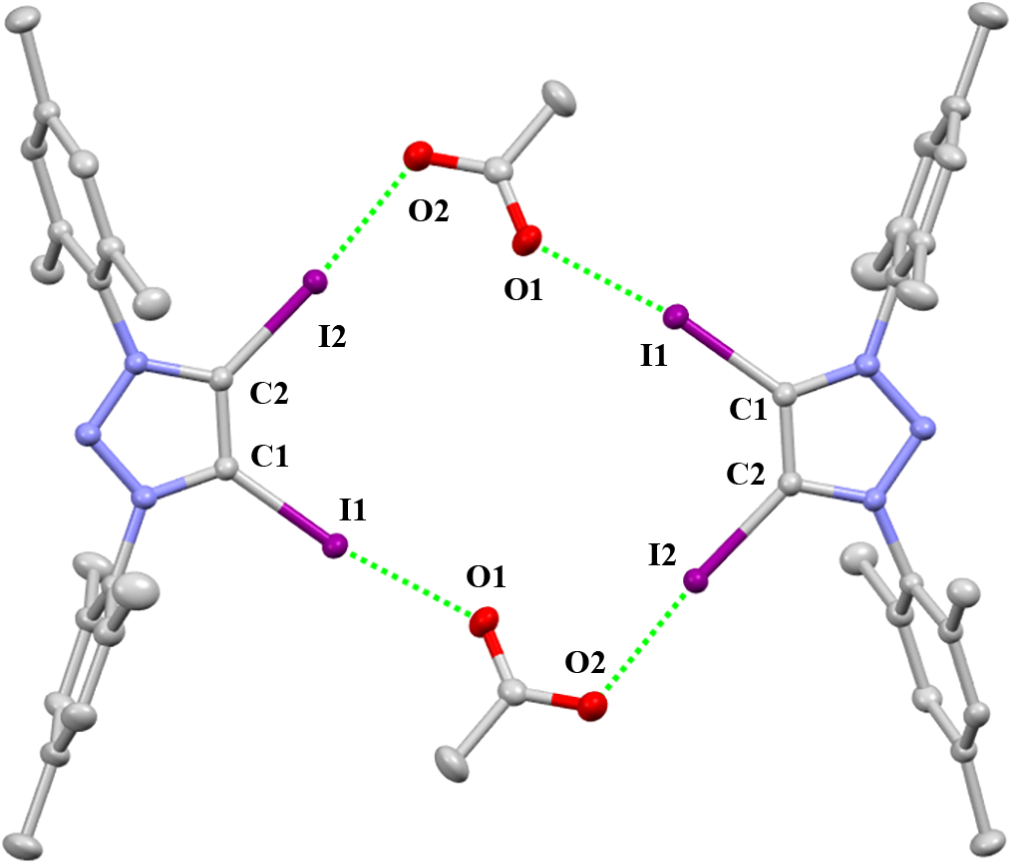
Packing structure of **2-OAc**. Hydrogen atoms and solvent molecules have been omitted for clarity.

The single crystal of **2-OAc** crystallizes in the monoclinic space group *P*2_1_/*c*. The package diagram shows a dimer which is almost a rectangle (Figure 4). As shown in Table 1, The C–I···O distances are 2.547(2) Å and 2.582(2) Å. The C–I···O angles are 174.60(7)° and 170.38(7)°. The R_XB_ values are 0.72 and 0.73.

**Figure 5:**
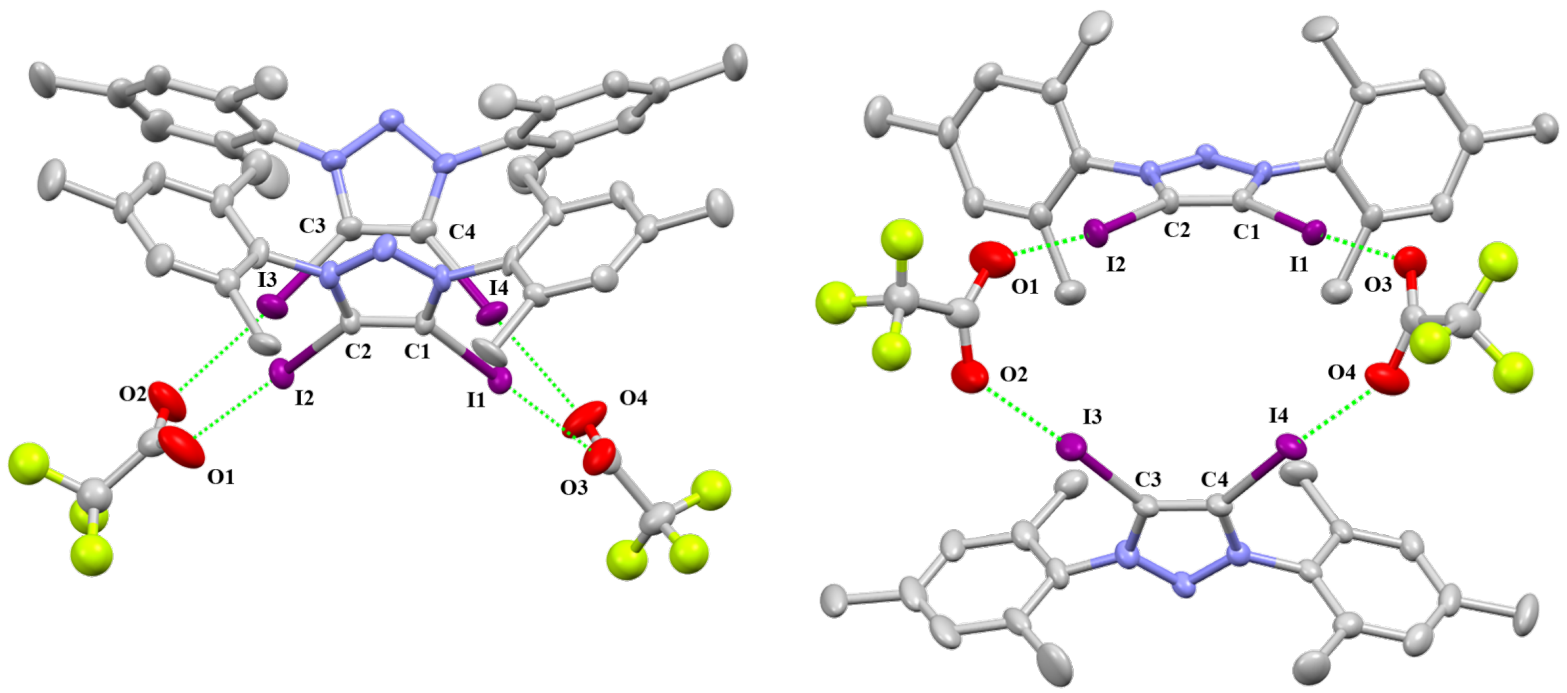
Packing structure of **2-TFA**. Hydrogen atoms and disorder of fluorine atoms have been omitted for clarity. Side view (left) and top view (right).

The single crystal of **2-TFA** crystallizes in the monoclinic space group *P*2_1_/*n*, but the packing structure of **2-TFA** is different**.** The package diagram shows that two cations and two acetates form a boat shape (Figure 5). The C–I···O distances are 2.631(8) Å, 2.739(8) Å, 2.666(6) Å and 2.68(1) Å. The C–I···O angles are 175.7(3)°, 172.7(3)°, 176.7(3)° and 176.1(3)°. The R_XB_ values are 0.77, 0.74 and 0.75 (Table 1).

**Figure 6:**
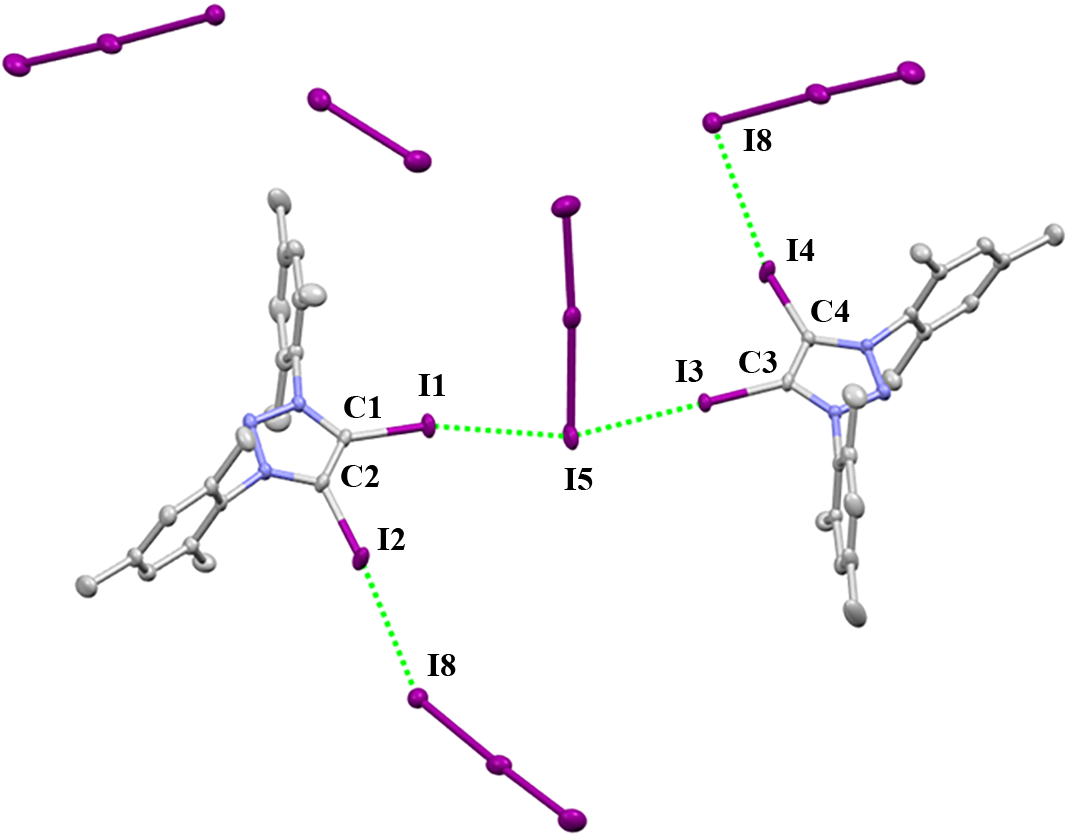
Packing structure of **2-I^.^1.5I_2_**. Hydrogen atoms have been omitted for clarity.

**Figure 7:**
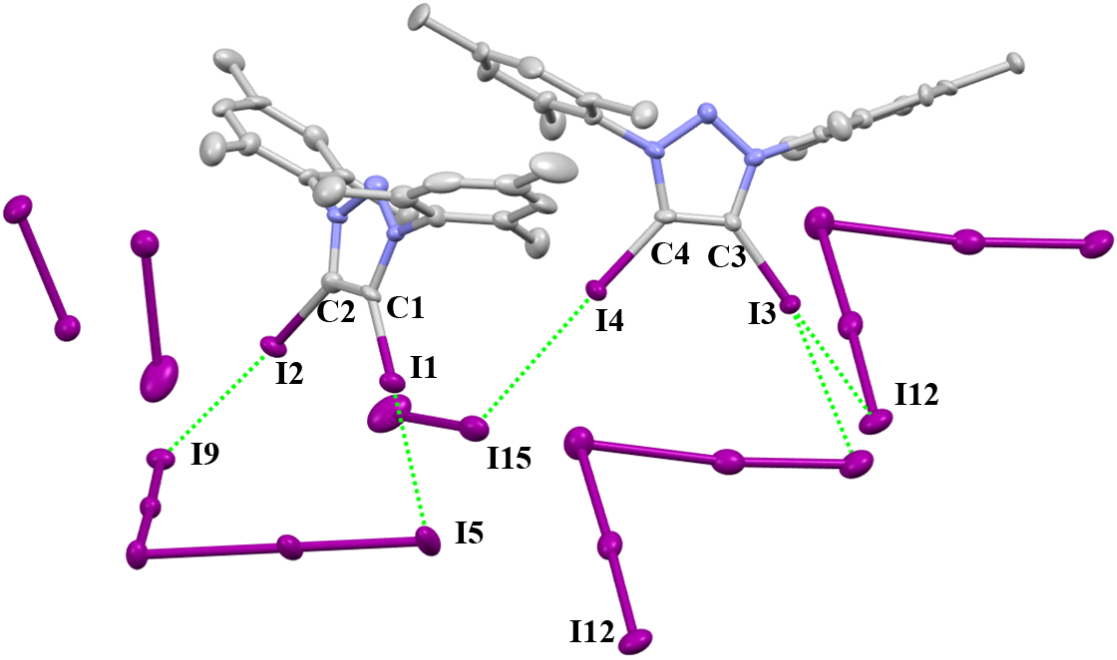
Packing structure of **2-I^.^3.5I_2_**. Hydrogen atoms have been omitted for clarity.

Triazolium polyiodide **2-I·1.5I_2_** was made by **2-I** with iodine and crystallizes in the monoclinic space group *P*2_1_/c. The crystal package diagram displays that a bent arrangement of XB donors are around the central iodine atom of the I_3_^−^ anion (Figure 6). **2-I·3.5I_2_** crystallizes in the monoclinic space group *P*2_1_/c (Figure 7). There are two I_3_^−^ and one I_2_ molecule in **2-I·1.5I_2_**, while two I_5_^−^ and three I_2_ molucules can be found in **2-I·3.5I_2_**.

**Table 1:** XB interactions of the crystals.

compound	interaction	I···X distance (Å)	R_XB_	angle C–I···X (deg)	C–I bond length (Å)

**2-I**	I1···I3	3.306(1)	0.81	C1–I1···I3 176.9(3)	C1–I1 2.101(9)
	I2···I3	3.300(1)	0.81	C2–I2···I3 176.2(3)	C2–I2 2.089(10)
**2-Br**	I1···Br1	3.107(2)	0.80	C1–I1···Br1 173.4(4)	C1–I1 2.10(1)
	I2···Br1	3.123(2)	0.80	C2–I2···Br1 177.1(4)	C2–I2 2.10(1)
**2-Cl**	I1···Cl1	2.960(8)	0.77	C1–I1···Cl1 176.8(6)	C1–I1 2.09(2)
	I2···Cl2	2.973(5)	0.77	C2–I2···Cl2 173.4(6)	C2–I2 2.05(2)
	I3···Cl2	2.975(7)	0.77	C3–I3···Cl2 176.2(6)	C3–I3 2.08(2)
	I4···Cl1	2.97(1)	0.77	C4–I4···Cl1 175.9(6)	C4–I4 2.09(3)
**2-OAc**	I1···O1	2.582(2)	0.73	C1–I1···O1 170.38(7)	C1–I1 2.085(2)
	I2···O2	2.547(2)	0.72	C2–I2···O2 174.60(7)	C2–I2 2.105(2)
**2-TFA**	I1···O3	2.644(8)	0.74	C1–I1···O3 175.3(3)	C1–I1 2.082(7)
	I2···O1	2.710(8)	0.77	C2–I2···O1 173.0(3)	C2–I2 2.059(7)
	I3···O2	2.677(6)	0.75	C3–I3···O2 177.0(3)	C3–I3 2.087(8)
	I4···O4	2.676(8)	0.75	C4–I4···O4 176.3(3)	C4–I4 2.072(7)
**2-I.1.5 I_2_**	I1···I5	3.482(2)	0.85	C1–I1···I5 167.48(3)	C1–I1 2.061(4)
	I2···I8	3.538(1)	0.87	C2–I2···I8 155.79(3)	C2–I2 2.062(4)
	I3···I5	3.394(1)	0.83	C3–I3···I5 176.97(2)	C3–I3 2.070(4)
**2-I.3.5 I_2_**	I1···I5	3.5454(7)	0.87	C1–I1···I5 175.8(2)	C1–I1 2.047(7)
	I2···I9	3.5829(7)	0.88	C2–I2···I9 174.2(2)	C2–I2 2.068(7)
	I4···I15	3.7356(7)	0.92	C4–I4···I15 177.8(2)	C4–I4 2.063(7)
	I3···I12	3.7237(8)	0.92	C3–I3···I12 149.2(2)	C3–I3 2.048(7)

**Figure 8:**
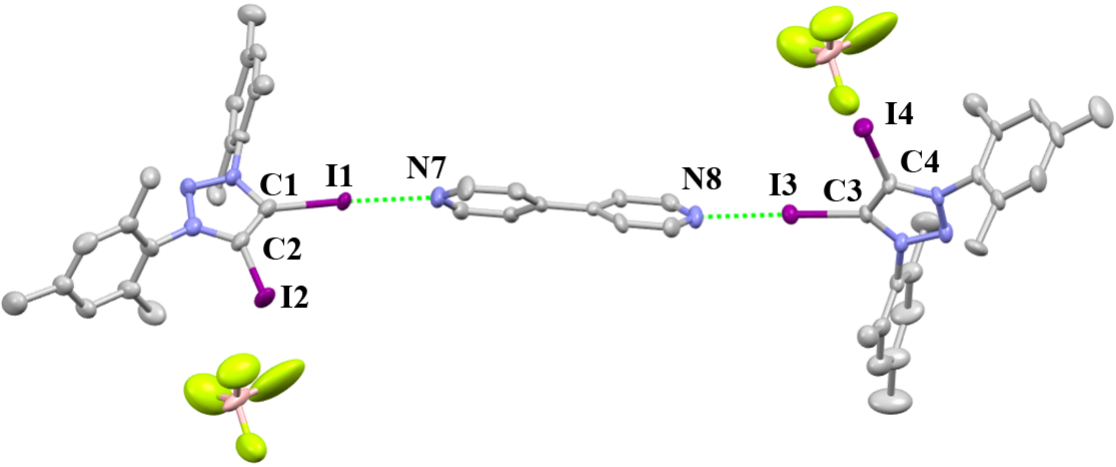
Packing structure of **2-BF_4_^.^0.5bpy**. Hydrogen atoms and dichloromethane have been omitted for clarity.

The XB interaction with neural halogen acceptors was also investigated. Diffusion of ether into the mixture of 4,4'-bipyridine (bpy) and **2-BF_4_** in dichloromethane leads to the crystallization of **2-BF_4_·0.5bpy** (Figure 8). It crystallizes in the monoclinic space group *P*2_1_/*c*. The acceptor 4,4'-bipyridine provides a complementary link for 1D chain formation. The C–I···N angles are 168.2(3)° and 173.4(4)°, close to linear, which is consistent with the high directionality of the interaction. The C–I···N distances are 2.599(9) Å and 2.580(9) Å. The R_XB_ value of C–I···N is 0.70. The C–I···F distances are 3.137(10) Å and 2.932(20) Å.

**Figure 9:**
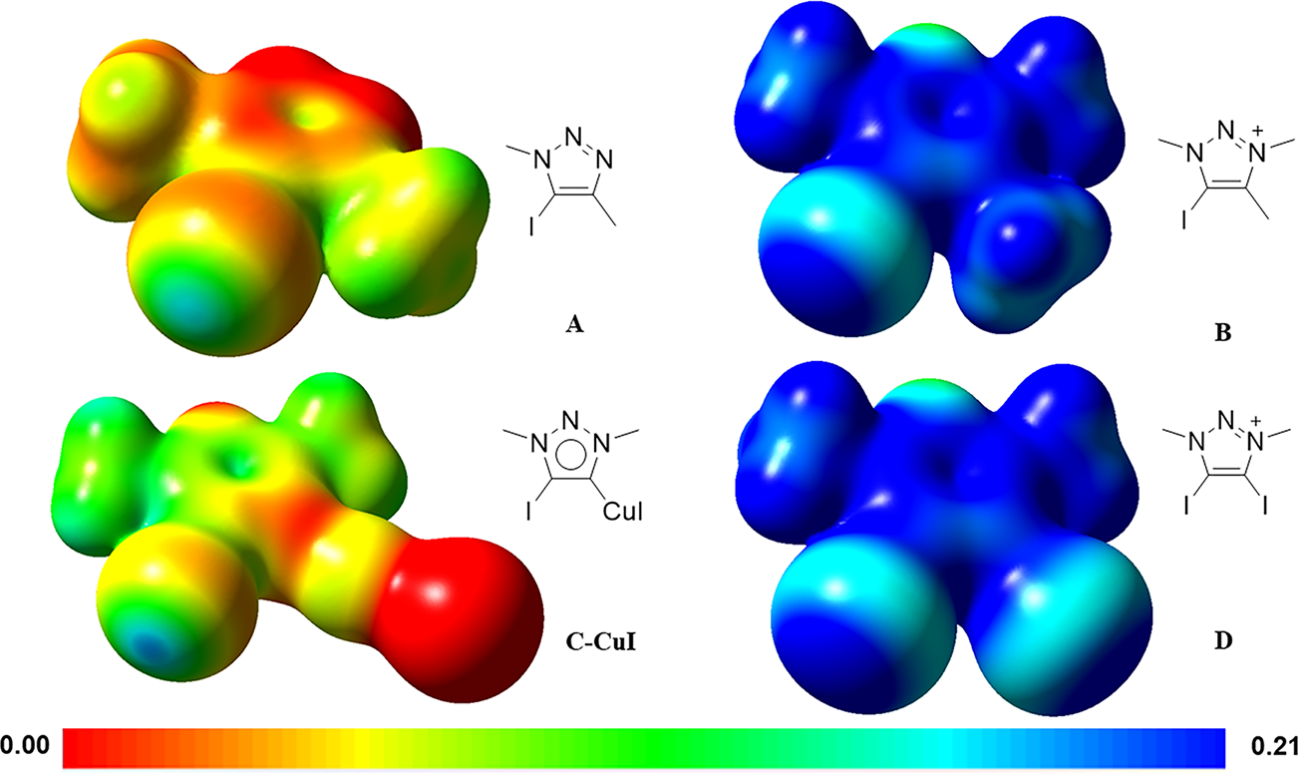
1,2,3-Triazole-based halogen model calculation: electrostatic potential surfaces mapped on total density (iso value 0.01). **C** was calculated using 1,2,3-triazolylidene copper iodide complex **C-CuI**.

To better understand the 1,2,3-triazole based XB donors, model 1,2,3-triazole **A**, 1,2,3-triazolium **B**, 1,2,3-triazolylidene complex **C-CuI** and diiodotriazolium **D** were calculated by DFT calculations (Figure 9). The calculation results show that σ holes in diiodotriazolium **D** are mainly located in the elongation of two C–I bonds. The DFT calculation also shows that σ hole of in diiodotriazolium **D** and 1,2,3-triazolium **B** are comparable, and much larger than the 1,2,3-triazole **A** and 1,2,3-triazolylidene complex **C-CuI** due to positive charge effect.

## Conclusion

In summary, we synthesized 4,5-diiodo-1,3-dimesityl-1,2,3-triazolium salts with different anions. When the anion is chloride, bromide or iodide, the crystal is a tetramer. Strong XB was observed in these forms. When the anion is changed to tetrafluoroborate, it takes Chinese lantern shape as a trimer. Triazolium trifluoroacetate and acetate exist as a dimer, while the former shows a boat conformation and the latter forms a rectangle conformation. Triazolium salts form a linear polymer with polyiodide. **2-BF_4_** forms co-crystals with 4,4'-bipyridine via halogen bonding. DFT calculation results show that the σ holes of 4,5-diiodo-1,2,3-triazolium is similar to the σ hole of 5-iodo-1,2,3-triazoliums salts.

## Experimental

### General

Unless otherwise noted, all reagents were obtained from commercial sources and used without further purification. All solvents were dried then stored over 4 Å molecular sieves prior to use. All syntheses were carried out under an atmosphere of dry nitrogen or in a glovebox. At the same time, the syntheses were performed under a standard Schlenk vacuum line. ^1^H NMR spectra were recorded on a Bruker Avance 400 MHz spectrometer. High-resolution mass spectra (HRMS) were acquired with a Thermo Scientific (Q-Exactive) instrument using electrospray ionization mode (ESI). Elemental analyses (C, H, N) were performed on Flash EA 1112 Analyzer.

### Synthesis

**2-I**: 4,5-Unsubstituted triazolium salt **1** (450 mg, 1 mmol), potassium *tert*-butoxide (250 mg, 2.2 mmol) and I_2_ (510 mg, 2 mmol) were added in a Schlenk tube under nitrogen, then THF (20 mL) was added at −78 °C. The mixture was stirred for 12 hours. After the evaporation of THF, dichloromethane (100 mL) was added and inorganic salts were removed by filtration. The pure product (521 mg) was obtained by evaporation of dichloromethane and washed with ether. The yield was 76%. A single crystal of **2-I** was obtained by slow diffusion of ether into a dimethylformamide solution due to the poor solubility. ^1^H NMR (400 MHz, CDCl_3_) δ 7.07 (s, 4H), 2.41 (s, 6H), 2.00 (s, 12H); ^13^C NMR (100 MHz, DMSO-*d*_6_) 143.2, 134.9, 131.9, 130.2, 111.8, 21.2, 17.1; HRMS (*m*/*z*): [M – I^−^]^+^ calcd for C_20_H_22_I_2_N_3_^+^, 557.9898; found, 557.9891; anal. calcd. for C_20_H_22_I_3_N_3_ (685.13): C, 35.06, H, 3.24, N, 6.13%; found: C, 35.41, H, 3.32, N, 6.06%.

**2-BF_4_**: AgBF_4_ (60 mg, 0.30 mmol) and **2-I** (205 mg, 0.30 mmol) were added in a Schlenk tube, then dichloromethane (5 mL) was added and the solution stirred under nitrogen in the dark for 6 h. After AgI was removed by filtration, the solution was washed with water. The pure product was obtained by evaporation of the dichloromethane phase (178 mg, 92% yield). A single crystal of **2-BF_4_** was obtained by slow diffusion of ether into a dichloromethane solution. ^1^H NMR (400 MHz, CDCl_3_) δ 7.12 (s, 4H), 2.40 (s, 6H), 2.02 (s, 12H); ^13^C NMR (100 MHz, CDCl_3_) δ 134.8, 134.2, 131.5, 130.3, 104.4, 12.4, 17.3; anal. calcd. for C_20_H_22_BF_4_I_2_N_3_ (645.03): C, 37.24, H, 3.44, N, 6.51%; found: C, 37.51, H, 3.52, N, 6.42%.

**2-Cl**: Potassium chloride (740 mg, 10 mmol) and **2-BF_4_** (120 mg, 0.19 mmol) were mixed in acetonitrile (30 mL) in a round bottom flask, then the mixture was stirred for 24 h under air. Then the acetonitrile was removed under reduced pressure. Dichloromethane (50 mL) was added and the excess potassium chloride was removed by filtration. Then the dichloromethane was removed by evaporation to give the final white product (**2-Cl**) (99 mg, 87% yield). ^1^H NMR (400 MHz, CDCl_3_) δ 7.05 (s, 4H), 2.39 (s, 6H), 1.98 (s, 12H); ^13^C NMR (100 MHz, CDCl_3_) δ 142.9, 134.2, 132.1, 130.1, 113.2, 21.4, 17.4; anal. calcd. for C_20_H_22_ClI_2_N_3_ (593.68): C, 40.46, H, 3.74, N, 7.08%; found: C, 40.81, H, 3.79, N, 6.96%. A single crystal of **2-Cl** was obtained by slow evaporation of a dichloromethane solution.

**2-Br**: Potassium bromide (1190 mg, 10 mmol) and **2-BF_4_** (128 mg, 0.2 mmol) were mixed in acetonitrile (30 mL) in a round bottom flask, then the mixture was stirred under air for 24 h. Then the acetonitrile was removed under reduced pressure, dichloromethane (70 mL) was added and the excess potassium bromide was removed by filtration. Then the dichloromethane was removed by evaporation to give the white product (**2-Br**) (112 mg, 88% yield). ^1^H NMR (400 MHz, CDCl_3_) δ 7.05 (s, 4H), 2.39 (s, 6H), 1.99 (s, 12H); ^13^C NMR (100 MHz, CDCl_3_) δ 143.0, 134.2, 132.1, 130.1, 113.8, 21.4, 17.4; anal. calcd. for C_20_H_22_BrI_2_N_3_ (638.13): C, 37.64, H, 3.48, N, 6.59%; found: C, 37.91, H, 3.65, N, 6.41%.

**2-OAc**: AgOAc (52 mg, 0.30 mmol) and **2-I** (205 mg, 0.30 mmol) were added in a Schlenk tube, then dichloromethane (6 mL) was added and the solution stirred in a glove box in the dark for 6 h. Then AgI was removed by filtration and the solution was washed with water (10 mL) to remove the excess of silver. The pure product was obtained by evaporation of dichloromethane solution (176 mg, 92%). ^1^H NMR (400 MHz, CDCl_3_) δ 7.00 (s, 4H), 2.32 (s, 6H), 1.92 (s, 12H), 1.75 (s, 3H); ^13^C NMR (100 MHz, CDCl_3_) δ 176.6, 142.6, 133.9, 131.8, 129.8, 110.8, 23.6, 21.2, 17.2; anal. calcd. for C_22_H_25_I_2_N_3_O_2_ (617.27): C, 42.81, H, 4.08, N, 6.81%; found: C, 42.96, H, 4.26, N, 6.72%. The single crystal of **2-OAc** was obtained by slow diffusion of *n*-pentane into a dichloromethane solution.

**2-TFA**: CF_3_COOAg (77 mg, 0.35 mmol) and **2-I** (205 mg, 0.3 mmol) were added in a Schlenk tube, then dichloromethane (6 mL) was added and the solution stirred in the glovebox for 6 h in the dark. Then AgI was removed by filtration and the solution was washed with water (5 mL) to remove the excess of silver. The pure product was obtained by evaporation of the dichloromethane solution (125 mg, 53%). ^1^H NMR (400 MHz, CDCl_3_) δ 7.08 (s, 4H), 2.40 (s, 6H), 2.00 (s, 12H); ^13^C NMR (100 MHz, CDCl_3_) δ 161.0 (q, *J* = 35.0 Hz), 143.3, 134.2, 131.9, 130.2, 116.5 (q, *J* = 297.2 Hz), 110.0, 21.4, 17.4; anal. calcd. for C_22_H_22_F_3_I_2_N_3_O_2_ (671.24): C, 39.37, H, 3.30, N, 6.26%; found: C, 39.51, H, 3.49, N, 6.18%.

**2-I^.^1.5I_2_**: **2-I** (35 mg, 0.05 mol) and I_2_ (25 mg, 0.1 mmol) were mixed in dichloromethane (6 mL) in a round bottom flask. The single crystals were obtained by slow diffusion of ether into a dichloromethane solution. Brown solid, 21 mg, 39% yield. ^1^H NMR (400 MHz, CDCl_3_) δ 7.20 (s, 4H), 2.47 (s, 6H), 2.09 (s, 12H); anal. calcd. for C_22_H_22_I_6_N_3_ (1065.84): C, 22.54, H, 2.08, N, 3.94%; found: 22.63, H, 2.19, N, 3.82%.

**2-I^.^3.5I_2_**: **2-I** (35 mg, 0.05 mol) and I_2_ (50 mg, 0.2 mmol) were mixed in dichloromethane (10 mL) in a round bottom flask. The single crystals were obtained by slow diffusion of ether into a dichloromethane solution, Brown solid, 63 mg, 81% yield. ^1^H NMR (400 MHz, CDCl_3_) δ 7.21 (s, 4H),2.48 (s, 6H), 2.10 (s, 12H); anal. calcd. for C_22_H_22_I_10_N_3_ (1073.46): C, 15.72, H, 1.41, N, 2.67%; found: C, 15.93, H, 1.66, N, 2.41%.

**2-BF_4_^.^0.5bpy**: **2-BF_4_** (65 mg, 0.1 mmol) and 4,4'-bipyridine (16 mg, 0.1 mmol) was mixed in dichloromethane (4 mL) in a round bottom flask. The single crystals were obtained by slow diffusion of ether into a dichloromethane solution. Colourless solid, 28 mg, 31% yield. ^1^H NMR (400 MHz, CDCl_3_) δ 8.60 (d, *J* = 6.1 Hz, 2H), 7.57 (d, *J* = 6.2 Hz, 2H), 7.11 (s, 4H), 2.39 (s, 6H), 2.02 (s, 12H); ^13^C NMR (100 MHz, CDCl_3_) δ 150.0, 145.7, 143.5, 134.0, 131.6, 130.2, 121.9, 107.6, 21.4, 17.2.

### X-ray diffraction measurements

Single crystal diffraction data for **2-I, 2-Br** and **2-Cl** were collected at 200 K using an IμS micro-focus sealed X-ray tube with Mo Kα radiation (λ = 0.71073 Å) on a Bruker D8 venture Kappa Duo diffractometer equipped with a PHOTON 100 detector. Low-temperature holding was achieved by a Cryostream Cooler (Oxford Cryosystems). Single crystal diffraction data for **2-OAc**, **2-TFA**, **2-BF_4_^.^0.5bpy**, **2-I^.^1.5I_2_** and **2-I^.^3.5I_2_** were collected at 150 K while **2-BF_4_** was collected at 298 K. All the data were collected 0.5 degree per step and using the ω scan mode. Frames were integrated using the Bruker SAINT [47] software. Semi-empirical absorption correction was applied with the SADABS program [48].

All the structures were solved by SHELXT [49] and refined by SHELXL [50] programs against |F|^2^ using all data following established refinement strategies [51] through olex2 [52]. Their packing diagrams were prepared by using Mercury [46].

### Computational details

All calculations were performed with the Gaussian 16 (G16) program package [53]. The DFT method using the M06-2X functional [54] relying on relativistic pseudo-potentials was used, namely the small core ECP46MWB [55] for I atoms. The C, H and N atoms were treated with a basis set of 6-311G** [56]. The Cu atom was treated with a basis set of LanL2DZ [57]. Geometry optimizations were performed without any constraints, and the frequency analysis confirmed that there were no image frequencies for these structures. Visualization of the electrostatic potential was performed using the Gauss View 6.0 package [58].

## Supporting Information

File 1 Crystallographic data, computational details, copies of ^1^H and ^13^C NMR spectra.

File 2 Crystallographic Information Files (CIF).
