# Supplementary material for: Halogen-bonding-induced diverse aggregation of 4,5-diiodo-1,2,3-triazolium salts with different anions
Source: Beilstein J Org Chem. 2020 Jan 13;16:78–87. doi: 10.3762/bjoc.16.10 (PMC7006493; doi:10.3762/bjoc.16.10)
Supplement: File 1 — Crystallographic data, computational details, copies of 1H and 13C NMR spectra. [file Beilstein_J_Org_Chem-16-78-s001.pdf]

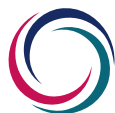

Supporting Information

for

Halogen-bonding-induced diverse aggregation of 4,5-diiodo-1,2,3-triazolium salts with different anions

Xingyu Xu, Shiqing Huang, Zengyu Zhang, Lei Cao and Xiaoyu Yan

Beilstein J. Org. Chem. **2020**, *16*, 78–87. doi:10.3762/bjoc.16.10

Crystallographic data, computational details, copies of ^1H and ^{13}C NMR spectra

Contents

1. Crystallography data	S1
2. Computational details.....	S19
3. Copies of ^1H and ^{13}C NMR spectra	S22

---

1. Crystallography data

Table S1 Crystal data and structure refinement for 2-I.

CCDC NO.	1923173
Identification code	mo_XXY2017120402_0m_a
Empirical formula	C ₂₀ H ₂₂ I ₃ N ₃
Formula weight	685.10
Temperature/K	200.0
Crystal system	tetragonal
Space group	$P \bar{4}2_1c$
a/Å	23.9419(10)
b/Å	23.9419(10)
c/Å	10.1217(3)
$\alpha/^\circ$	90
$\beta/^\circ$	90
$\gamma/^\circ$	90
Volume/Å ³	5801.9(5)
Z	8
$\rho_{\text{calc}}/\text{cm}^3$	1.569
μ/mm^{-1}	3.239
F(000)	2576.0
Crystal size/mm ³	0.38 × 0.31 × 0.23
Radiation	MoK α (λ = 0.71073)
2 Θ range for data collection/ $^\circ$	4.37 to 58.272
Index ranges	-32 ≤ h ≤ 24, -29 ≤ k ≤ 24, -11 ≤ l ≤ 13
Reflections collected	20762
Independent reflections	7767 [R_{int} = 0.0533, R_{sigma} = 0.0845]
Data/restraints/parameters	7767/9/217
Goodness-of-fit on F ²	1.120
Final R indexes [$I \geq 2\sigma(I)$]	R_1 = 0.0613, wR_2 = 0.1361
Final R indexes [all data]	R_1 = 0.1114, wR_2 = 0.1531
Largest diff. peak/hole / e Å ⁻³	1.38/-0.93
Flack parameter	-0.03(2)

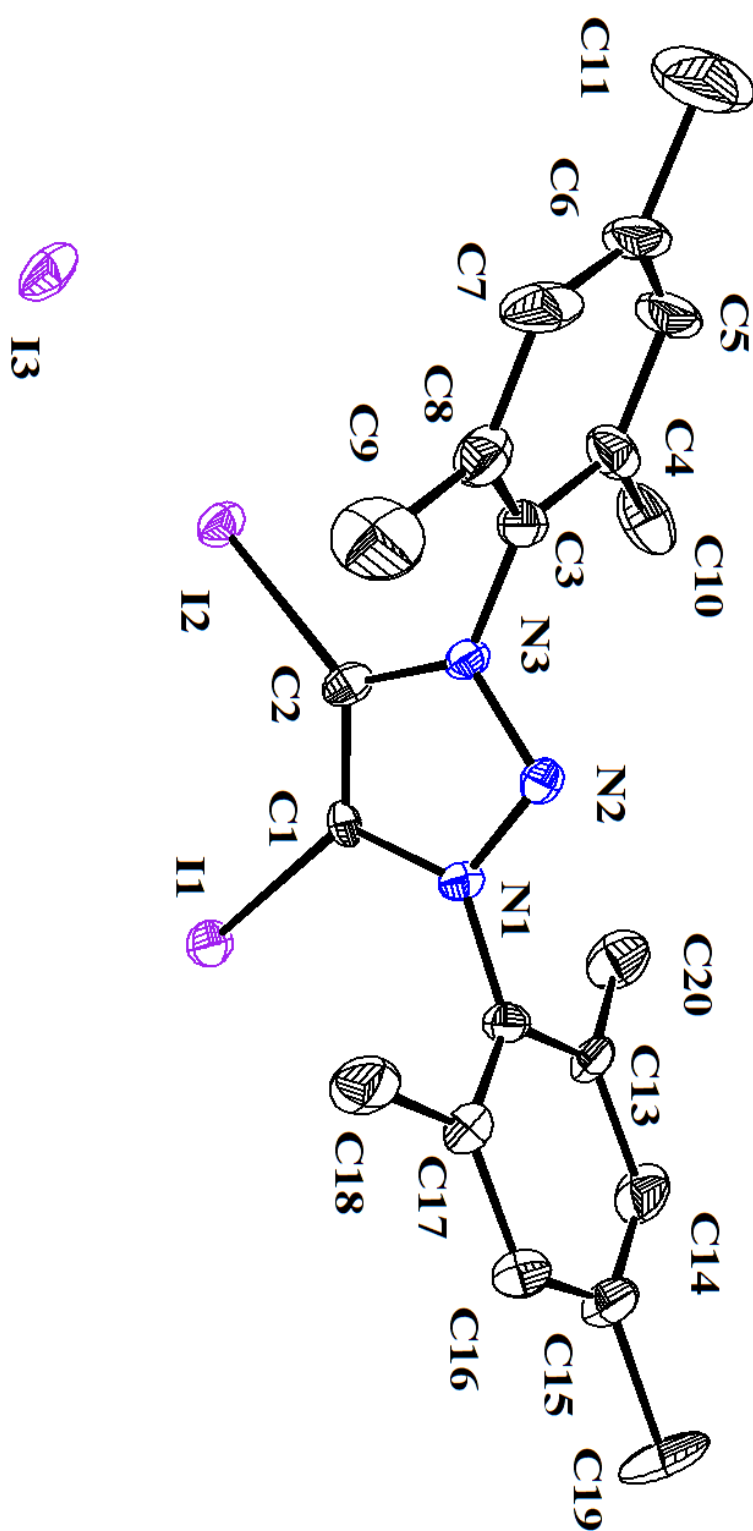

Figure S1 Thermal ellipsoid plot (ellipsoid contour 30%) for **2-I**. Hydrogen atoms are omitted for clarity.

Table S2 Crystal data and structure refinement for 2-Br.

CCDC NO.	1923174
Identification code	mo_xxy201809201_0m
Empirical formula	C ₂₀ H ₂₂ BrI ₂ N ₃
Formula weight	638.11
Temperature/K	200.0
Crystal system	tetragonal
Space group	$I \bar{4}$
a/Å	23.5982(13)
b/Å	23.5982(13)
c/Å	9.8455(6)
$\alpha/^\circ$	90
$\beta/^\circ$	90
$\gamma/^\circ$	90
Volume/Å ³	5482.7(7)
Z	8
$\rho_{\text{calc}}/\text{cm}^3$	1.546
μ/mm^{-1}	3.759
F(000)	2432.0
Crystal size/mm ³	0.37 × 0.36 × 0.29
Radiation	MoK α ($\lambda = 0.71073$)
2 Θ range for data collection/ $^\circ$	4.482 to 52.978
Index ranges	-33 ≤ h ≤ 31, -34 ≤ k ≤ 34, -14 ≤ l ≤ 14
Reflections collected	20093
Independent reflections	8644 [$R_{\text{int}} = 0.0536$, $R_{\text{sigma}} = 0.0824$]
Data/restraints/parameters	8644/6/218
Goodness-of-fit on F ²	1.074
Final R indexes [$I \geq 2\sigma(I)$]	$R_1 = 0.0542$, $wR_2 = 0.1425$
Final R indexes [all data]	$R_1 = 0.0790$, $wR_2 = 0.1553$
Largest diff. peak/hole / e Å ⁻³	1.90/-1.27
Flack parameter	0.057(13)

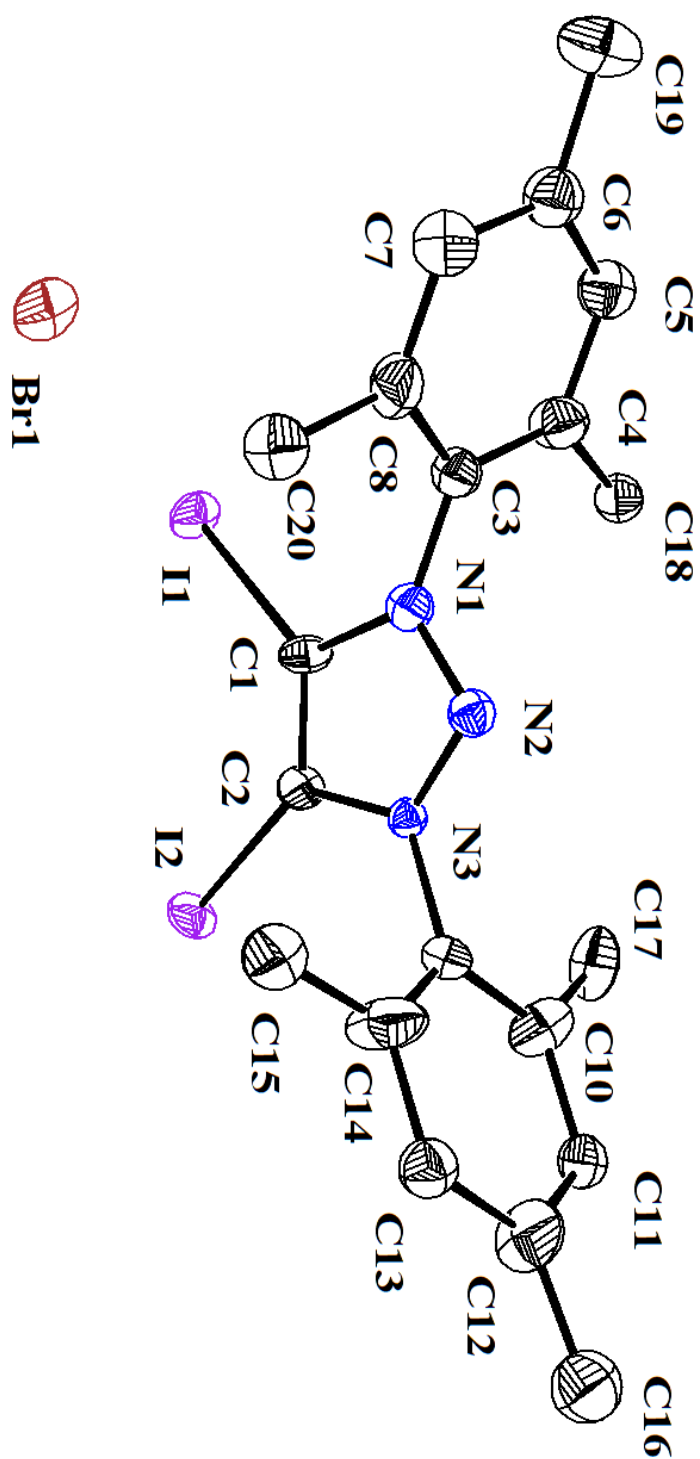

Figure S2 Thermal ellipsoid plot (ellipsoid contour 30%) for **2-Br**. Hydrogen atoms are omitted for clarity.

Table S3 Crystal data and structure refinement for 2-Cl.

CCDC NO.	1923175
Identification code	mo_XXY318_0m_a
Empirical formula	C ₂₀ H ₂₂ ClI ₂ N ₃
Formula weight	593.65
Temperature/K	200.0
Crystal system	monoclinic
Space group	C2
a/Å	32.770(4)
b/Å	9.7269(13)
c/Å	23.215(3)
$\alpha/^\circ$	90
$\beta/^\circ$	134.483(3)
$\gamma/^\circ$	90
Volume/Å ³	5279.3(12)
Z	8
$\rho_{\text{calc}}/\text{cm}^3$	1.494
μ/mm^{-1}	2.491
F(000)	2288.0
Crystal size/mm ³	0.16 × 0.15 × 0.15
Radiation	MoK α (λ = 0.71073)
2 Θ range for data collection/ $^\circ$	4.536 to 55.164
Index ranges	-35 ≤ h ≤ 42, -11 ≤ k ≤ 12, -30 ≤ l ≤ 27
Reflections collected	20706
Independent reflections	9877 [R_{int} = 0.0557, R_{sigma} = 0.0904]
Data/restraints/parameters	9877/301/430
Goodness-of-fit on F ²	1.054
Final R indexes [$I \geq 2\sigma(I)$]	R_1 = 0.0591, wR_2 = 0.1166
Final R indexes [all data]	R_1 = 0.0893, wR_2 = 0.1272
Largest diff. peak/hole / e Å ⁻³	1.62/-1.72
Flack parameter	0.009(19)

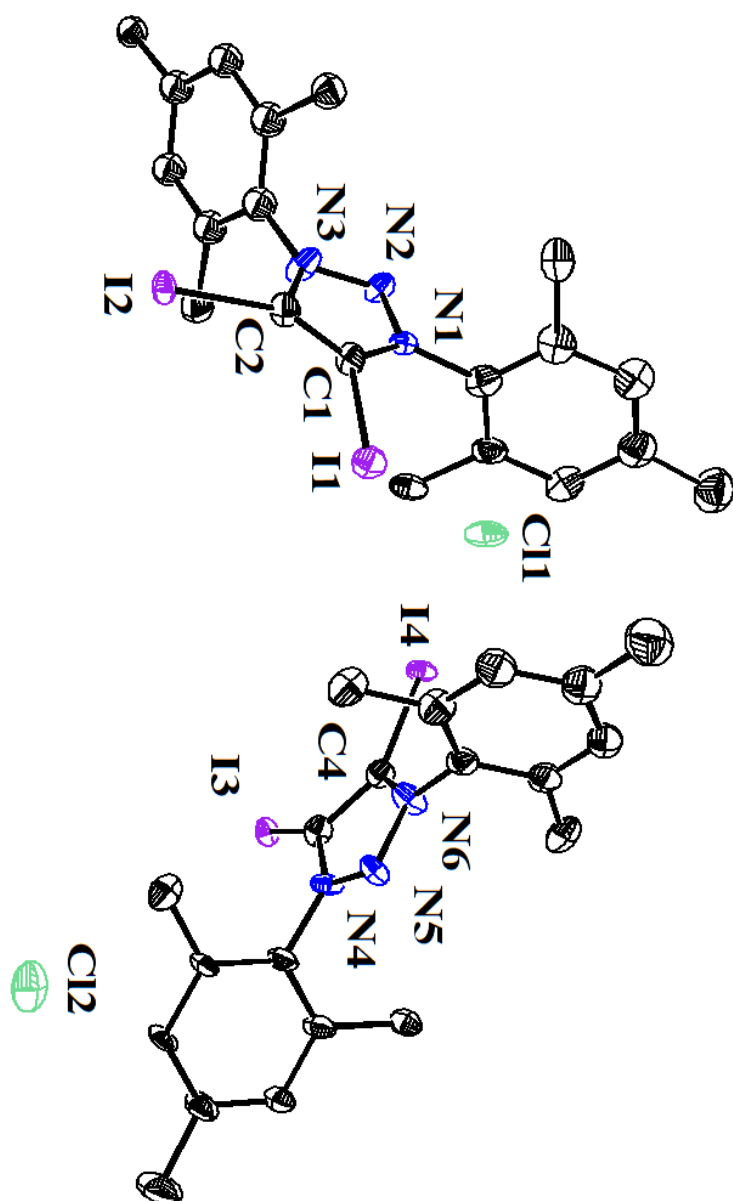

Figure S3 Thermal ellipsoid plot (ellipsoid contour 30%) for **2-Cl**. Hydrogen atoms are omitted for clarity.

Table S4 Crystal data and structure refinement for 2-OAc.

CCDC NO.	1923176
Identification code	mo_XXY201808311_0ma_a
Empirical formula	C ₂₃ H ₂₇ Cl ₂ I ₂ N ₃ O ₂
Formula weight	702.17
Temperature/K	150.0
Crystal system	monoclinic
Space group	<i>P</i> 2 ₁ / <i>c</i>
<i>a</i> /Å	11.9179(10)
<i>b</i> /Å	27.124(2)
<i>c</i> /Å	8.6997(7)
α /°	90
β /°	107.515(3)
γ /°	90
Volume/Å ³	2681.9(4)
<i>Z</i>	4
ρ_{calc} /cm ³	1.739
μ /mm ⁻¹	2.568
<i>F</i> (000)	1368.0
Crystal size/mm ³	0.46 × 0.35 × 0.31
Radiation	MoK α (λ = 0.71073)
2 Θ range for data collection/°	4.676 to 61.094
Index ranges	-17 ≤ <i>h</i> ≤ 12, -38 ≤ <i>k</i> ≤ 38, -12 ≤ <i>l</i> ≤ 12
Reflections collected	37189
Independent reflections	8206 [<i>R</i> _{int} = 0.0316, <i>R</i> _{sigma} = 0.0255]
Data/restraints/parameters	8206/0/296
Goodness-of-fit on <i>F</i> ²	1.117
Final <i>R</i> indexes [<i>I</i> ≥ 2 σ (<i>I</i>)]	<i>R</i> ₁ = 0.0258, <i>wR</i> ₂ = 0.0563
Final <i>R</i> indexes [all data]	<i>R</i> ₁ = 0.0321, <i>wR</i> ₂ = 0.0582
Largest diff. peak/hole / e Å ⁻³	1.16/-0.96

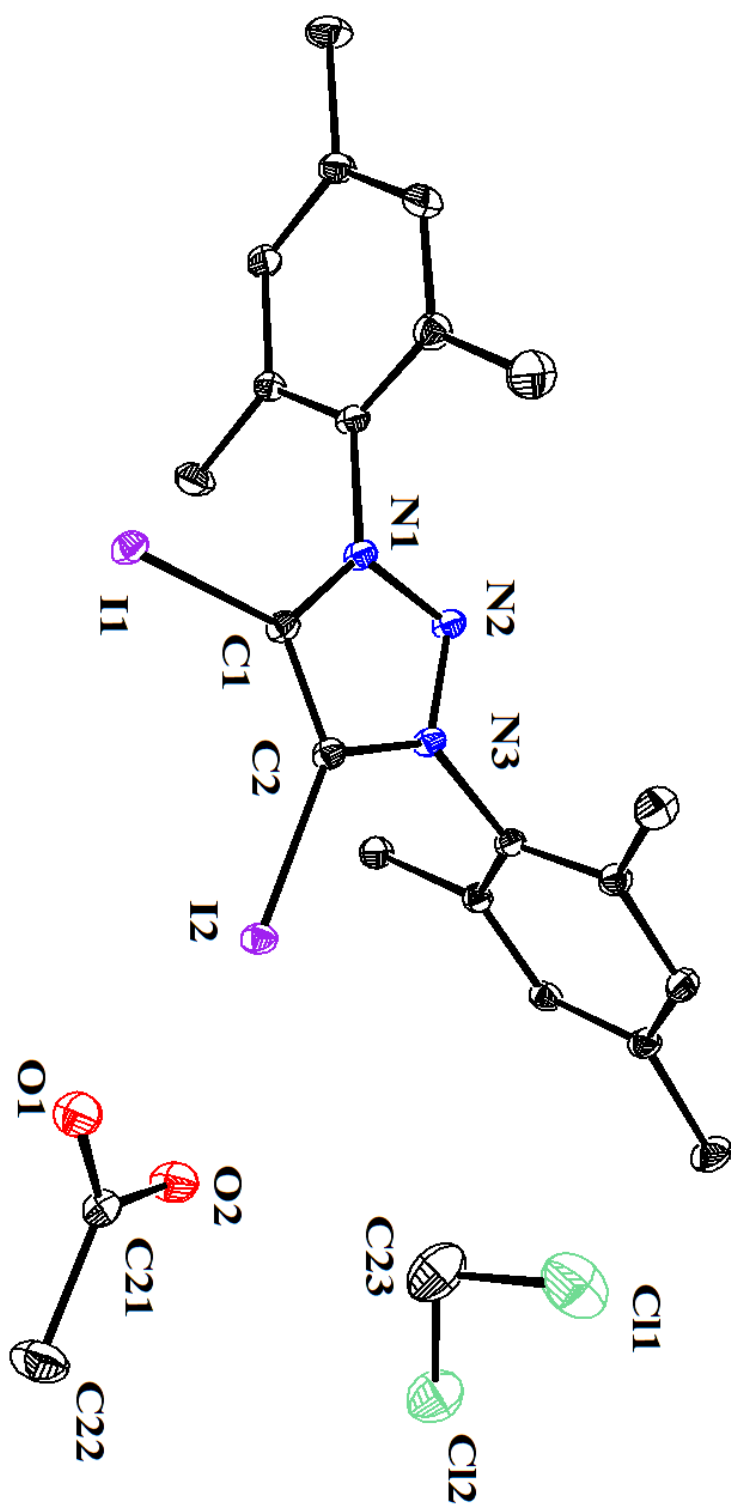

Figure S4 Thermal ellipsoid plot (ellipsoid contour 30%) for **2-OAc**. Hydrogen atoms are omitted for clarity.

Table S5 Crystal data and structure refinement for 2-TFA.

CCDC NO.	1923177
Identification code	mo_XXY4251_0m_a
Empirical formula	C ₂₂ H ₂₂ F ₃ I ₂ N ₃ O ₂
Formula weight	671.22
Temperature/K	150.0
Crystal system	monoclinic
Space group	<i>P</i> 2 ₁ / <i>n</i>
<i>a</i> /Å	22.3708(19)
<i>b</i> /Å	11.2189(12)
<i>c</i> /Å	22.842(2)
α /°	90
β /°	117.810(3)
γ /°	90
Volume/Å ³	5070.7(9)
<i>Z</i>	8
$\rho_{\text{calc}}/\text{cm}^3$	1.758
μ/mm^{-1}	2.525
<i>F</i> (000)	2592.0
Crystal size/mm ³	0.23 × 0.19 × 0.15
Radiation	MoK α (λ = 0.71073)
2 Θ range for data collection/°	5.036 to 55.072
Index ranges	-29 ≤ <i>h</i> ≤ 26, -14 ≤ <i>k</i> ≤ 14, -29 ≤ <i>l</i> ≤ 29
Reflections collected	49462
Independent reflections	11611 [<i>R</i> _{int} = 0.0535, <i>R</i> _{sigma} = 0.0501]
Data/restraints/parameters	11611/630/597
Goodness-of-fit on <i>F</i> ²	1.132
Final <i>R</i> indexes [<i>I</i> ≥ 2 σ (<i>I</i>)]	<i>R</i> ₁ = 0.0914, <i>wR</i> ₂ = 0.2130
Final <i>R</i> indexes [all data]	<i>R</i> ₁ = 0.1111, <i>wR</i> ₂ = 0.2216
Largest diff. peak/hole / e Å ⁻³	3.58/-1.87

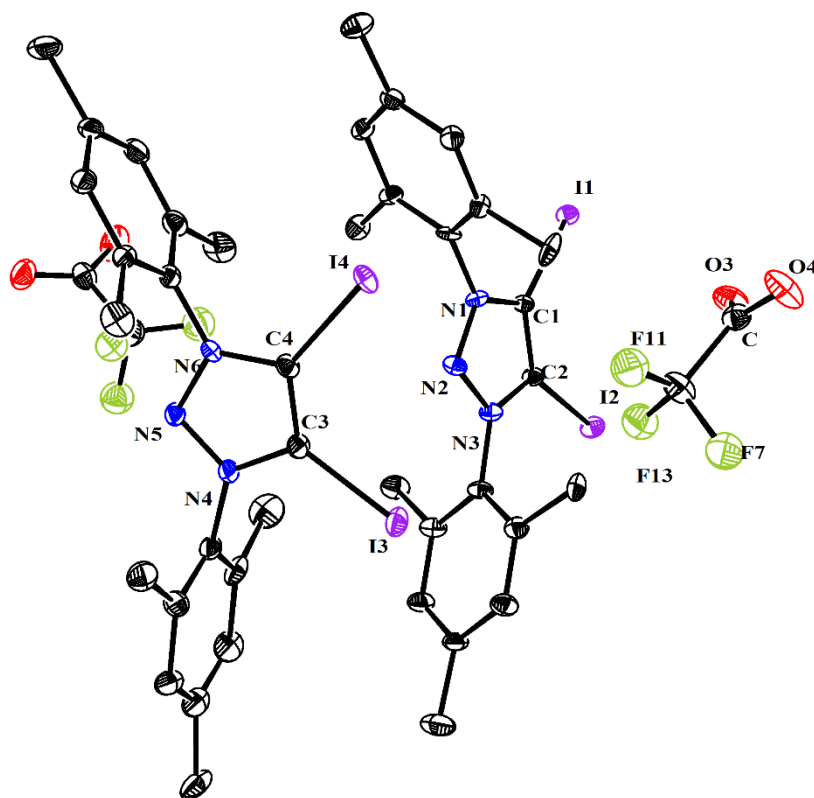

Figure S5 Thermal ellipsoid plot (ellipsoid contour 30%) for **2-TFA**. Hydrogen atoms are omitted for clarity.

Table S6 Crystal data and structure refinement for 2-BF₄.

CCDC NO.	1923178
Identification code	mo_XXY2017111702_0m_a
Empirical formula	C ₂₀ H ₂₂ BF ₄ I ₂ N ₃
Formula weight	645.01
Temperature/K	297.85
Crystal system	triclinic
Space group	$P \bar{1}$
a/Å	15.734(3)
b/Å	16.150(4)
c/Å	18.906(4)
$\alpha/^\circ$	109.625(6)
$\beta/^\circ$	90.918(6)
$\gamma/^\circ$	118.792(5)
Volume/Å ³	3872.4(15)
Z	6
$\rho_{\text{calc}}/\text{cm}^3$	1.660
μ/mm^{-1}	2.475
F(000)	1860.0
Crystal size/mm ³	0.26 × 0.23 × 0.21
Radiation	MoK α ($\lambda = 0.71073$)
2 Θ range for data collection/ $^\circ$	4.498 to 49.998
Index ranges	-18 ≤ h ≤ 18, -17 ≤ k ≤ 19, -22 ≤ l ≤ 21
Reflections collected	40817
Independent reflections	13582 [$R_{\text{int}} = 0.0987$, $R_{\text{sigma}} = 0.1191$]
Data/restraints/parameters	13582/79/829
Goodness-of-fit on F ²	1.045
Final R indexes [$I \geq 2\sigma(I)$]	$R_1 = 0.0921$, $wR_2 = 0.2475$
Final R indexes [all data]	$R_1 = 0.1692$, $wR_2 = 0.2991$
Largest diff. peak/hole / e Å ⁻³	1.71/-1.19

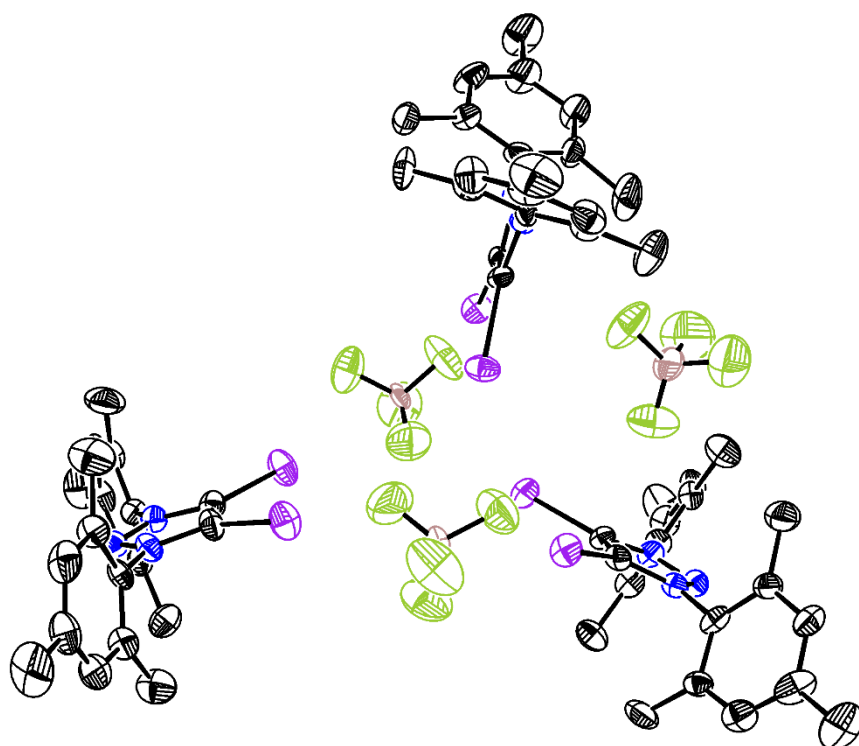

Figure S6 Thermal ellipsoid plot (ellipsoid contour 30%) for **2-BF₄**. Hydrogen atoms are omitted for clarity.

Table S7 Crystal data and structure refinement for 2-I-1.5I₂.

CCDC NO.	1923179
Identification code	mo_XXY18922_0m_a
Empirical formula	C ₄₀ H ₄₄ I ₁₂ N ₆
Formula weight	2131.61
Temperature/K	150.0
Crystal system	monoclinic
Space group	<i>P</i> 2 ₁ / <i>c</i>
<i>a</i> /Å	16.791(6)
<i>b</i> /Å	28.467(11)
<i>c</i> /Å	12.617(5)
α /°	90
β /°	107.753(15)
γ /°	90
Volume/Å ³	5744(4)
<i>Z</i>	4
$\rho_{\text{calc}}/\text{cm}^3$	2.465
μ/mm^{-1}	6.502
<i>F</i> (000)	3848.0
Crystal size/mm ³	0.45 × 0.39 × 0.35
Radiation	MoK α (λ = 0.71073)
2 Θ range for data collection/°	4.436 to 55.084
Index ranges	-21 ≤ <i>h</i> ≤ 21, -36 ≤ <i>k</i> ≤ 36, -16 ≤ <i>l</i> ≤ 16
Reflections collected	95836
Independent reflections	13122 [<i>R</i> _{int} = 0.0547, <i>R</i> _{sigma} = 0.0322]
Data/restraints/parameters	13122/0/555
Goodness-of-fit on <i>F</i> ²	1.088
Final <i>R</i> indexes [<i>I</i> ≥ 2 σ (<i>I</i>)]	<i>R</i> ₁ = 0.0310, <i>wR</i> ₂ = 0.0645
Final <i>R</i> indexes [all data]	<i>R</i> ₁ = 0.0394, <i>wR</i> ₂ = 0.0673
Largest diff. peak/hole / e Å ⁻³	2.14/-2.36

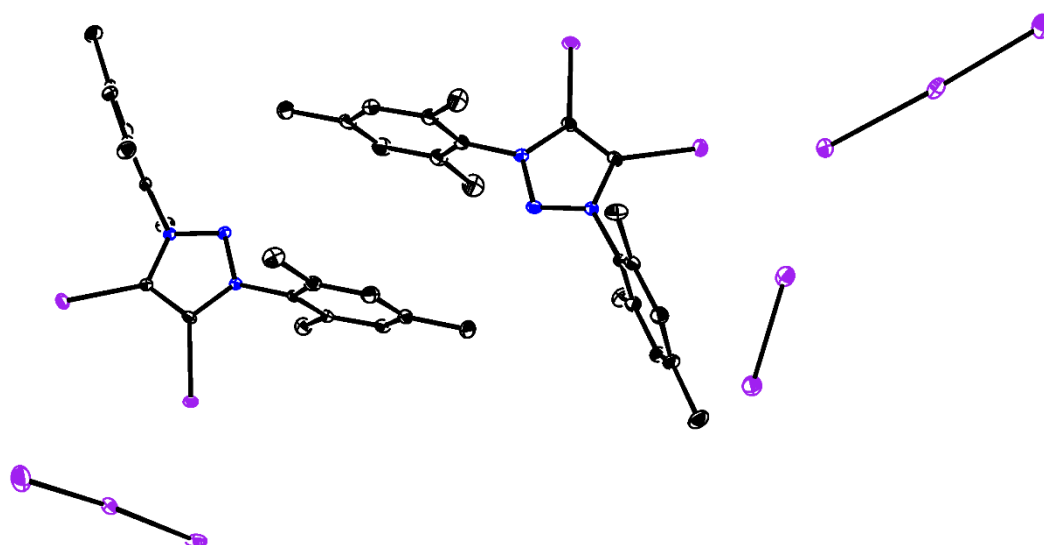

Figure S7 Thermal ellipsoid plot (ellipsoid contour 30%) for **2-I·1.5I₂**. Hydrogen atoms are omitted for clarity.

Table S8 Crystal data and structure refinement for 2-I-3.5I₂.

CCDC NO.	1923180
Identification code	mo_XXY18934_0m_a
Empirical formula	C ₄₀ H ₄₄ I ₂₀ N ₆
Formula weight	3146.81
Temperature/K	149.98
Crystal system	monoclinic
Space group	<i>P</i> 2 ₁ / <i>c</i>
<i>a</i> /Å	12.2054(5)
<i>b</i> /Å	28.5826(15)
<i>c</i> /Å	20.7771(11)
α /°	90
β /°	99.232(2)
γ /°	90
Volume/Å ³	7154.5(6)
<i>Z</i>	4
ρ_{calc} /cm ³	2.921
μ /mm ⁻¹	8.677
<i>F</i> (000)	5544.0
Crystal size/mm ³	0.53 × 0.43 × 0.42
Radiation	MoK α (λ = 0.71073)
2 Θ range for data collection/°	4.422 to 55.058
Index ranges	-15 ≤ <i>h</i> ≤ 15, -37 ≤ <i>k</i> ≤ 37, -26 ≤ <i>l</i> ≤ 22
Reflections collected	56334
Independent reflections	16405 [<i>R</i> _{int} = 0.0579, <i>R</i> _{sigma} = 0.0614]
Data/restraints/parameters	16405/0/626
Goodness-of-fit on <i>F</i> ²	1.012
Final <i>R</i> indexes [<i>I</i> ≥ 2 σ (<i>I</i>)]	<i>R</i> ₁ = 0.0430, <i>wR</i> ₂ = 0.0900
Final <i>R</i> indexes [all data]	<i>R</i> ₁ = 0.0673, <i>wR</i> ₂ = 0.0997
Largest diff. peak/hole / e Å ⁻³	2.02/-2.68

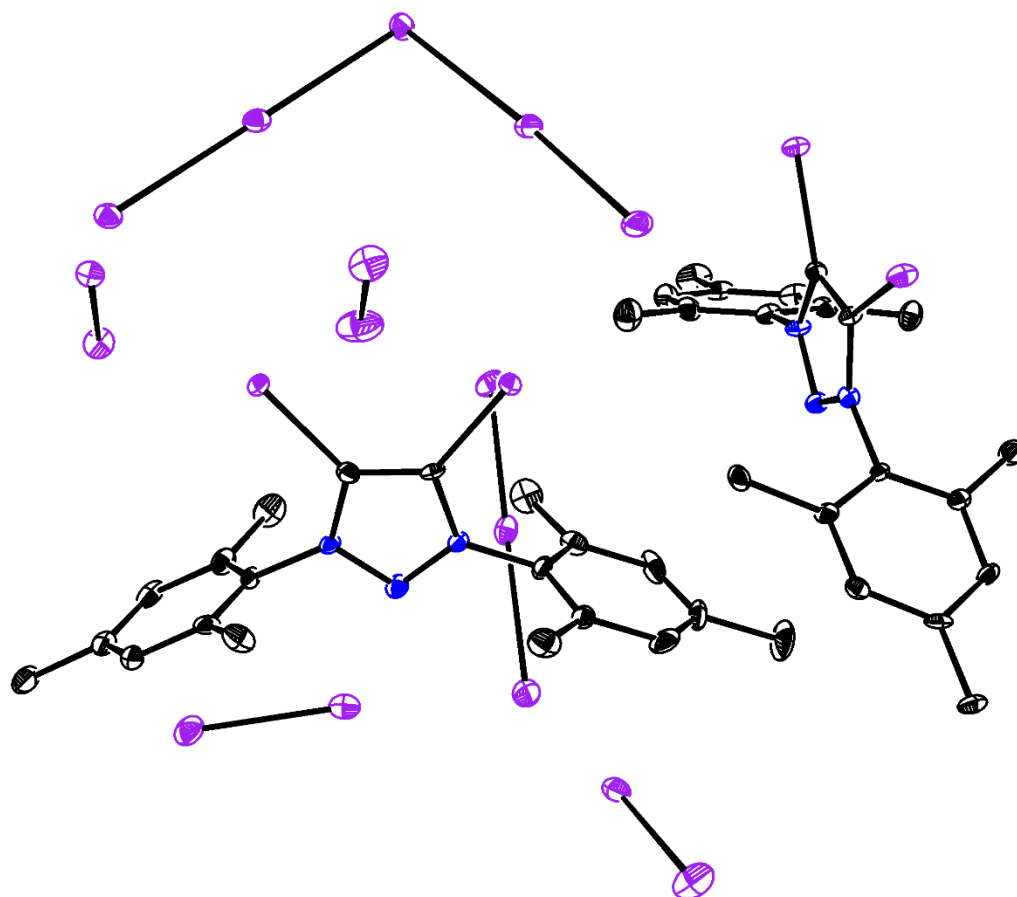

Figure S8 Thermal ellipsoid plot (ellipsoid contour 30%) for **2-I_{3.5}I₂**. Hydrogen atoms are omitted for clarity.

Table S9 Crystal data and structure refinement for 2-BF₄.0.5 bpy

CCDC NO.	1923181
Identification code	mo_XXY1891_0m_a
Empirical formula	C ₅₄ H ₆₀ B ₂ Cl ₈ F ₈ I ₄ N ₈
Formula weight	1785.92
Temperature/K	149.97
Crystal system	monoclinic
Space group	<i>P</i> 2 ₁ / <i>c</i>
<i>a</i> /Å	18.1509(13)
<i>b</i> /Å	18.7643(13)
<i>c</i> /Å	20.4941(16)
α /°	90
β /°	92.369(2)
γ /°	90
Volume/Å ³	6974.1(9)
<i>Z</i>	4
$\rho_{\text{calc}}/\text{cm}^3$	1.701
μ/mm^{-1}	2.156
<i>F</i> (000)	3480.0
Crystal size/mm ³	0.42 × 0.35 × 0.31
Radiation	MoK α (λ = 0.71073)
2 Θ range for data collection/°	4.488 to 55.088
Index ranges	-23 ≤ <i>h</i> ≤ 23, -24 ≤ <i>k</i> ≤ 24, -25 ≤ <i>l</i> ≤ 26
Reflections collected	67328
Independent reflections	15951 [<i>R</i> _{int} = 0.0439, <i>R</i> _{sigma} = 0.0404]
Data/restraints/parameters	15951/54/809
Goodness-of-fit on <i>F</i> ²	1.152
Final <i>R</i> indexes [<i>I</i> ≥ 2 σ (<i>I</i>)]	<i>R</i> ₁ = 0.0965, <i>wR</i> ₂ = 0.2020
Final <i>R</i> indexes [all data]	<i>R</i> ₁ = 0.1121, <i>wR</i> ₂ = 0.2088
Largest diff. peak/hole / e Å ⁻³	3.01/-2.19

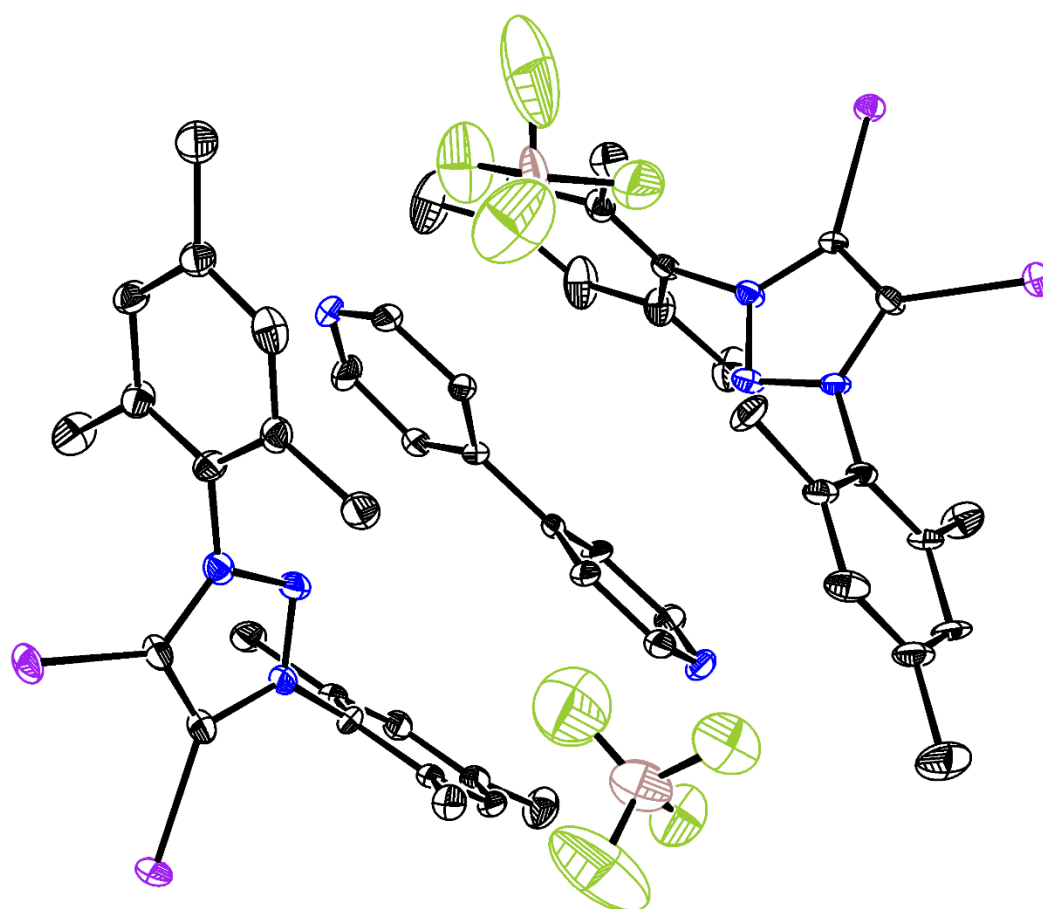

Figure S9 Thermal ellipsoid plot (ellipsoid contour 30%) for **2-BF₄.0.5 bpy**. Hydrogen atoms and solvents CH₂Cl₂ are omitted for clarity.

2. Computational details

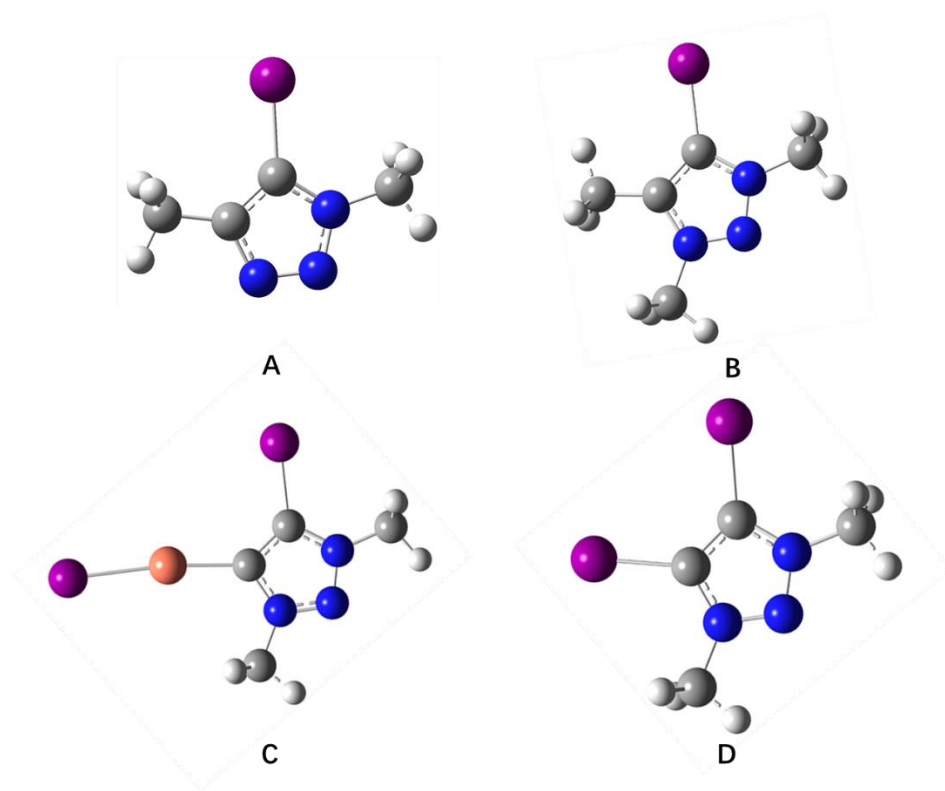

Figure 1. The optimized structures of **A**, **B**, **C-CuI**, and **D** at m06-2x level with basis of 6-311G (d, p) for H, C, N atoms, LanL2DZ for Cu atom and MWB46 for I atoms.

Table 1. Thermodynamic data of **A**, **B**, **C**, and **D**.

Compounds	E(Thermal)<br>KCal/Mol	CV<br>Cal/Mol-Kelvin	S<br>Cal/Mol-Kelvin
**A**	71.378	27.800	92.641
**B**	98.483	34.422	102.835
**C-CuI**	75.209	36.805	118.913
**D**	74.261	32.711	103.736

Cartesian coordinates

A

C	-1.49248200	1.07009900	-0.00003200
C	-0.59459600	0.02853000	-0.00024400
N	-2.64391500	-0.76996200	0.00035500
N	-1.34920800	-1.09362300	0.00001000
N	-2.73438400	0.52153200	0.00032400
I	1.48869000	0.04139300	0.00006000
C	-0.93881800	-2.48398100	-0.00034300
H	-1.85134100	-3.07477000	-0.00027500
H	-0.35060500	-2.70242100	-0.89199200

H	-0.35023400	-2.70275200	0.89098300
C	-1.25172600	2.54017200	-0.00030300
H	-0.69314600	2.85070700	0.88534600
H	-0.68406400	2.84873900	-0.88082300
H	-2.21288600	3.05213100	-0.00572500
**B**			
C	-1.29520300	-0.80273300	0.00006900
C	-0.25223900	0.09754800	0.00011200
N	-2.13437700	1.25782700	-0.00006800
N	-0.83296500	1.32830900	0.00006400
I	1.79771500	-0.23137600	-0.00005300
C	-0.17901100	2.64044100	0.00018000
H	0.43511000	2.72768000	-0.89460100
H	-0.96706200	3.38811800	0.00051900
H	0.43553600	2.72730600	0.89470200
N	-2.40717000	-0.01808200	-0.00006800
C	-3.80350800	-0.46097100	-0.00020500
H	-3.98790500	-1.05293000	-0.89520700
H	-3.98804800	-1.05317100	0.89463000
H	-4.42458200	0.43047000	-0.00016800
C	-1.33011000	-2.28708100	0.00026000
H	-1.84330900	-2.66391400	-0.88685500
H	-0.31353100	-2.67657500	0.00016300
H	-1.84307500	-2.66364300	0.88763100
**C-CuI**			
C	0.66821400	0.96996100	0.00119800
C	1.93910500	0.42249500	0.00000700
N	2.24901900	2.60245500	-0.00304700
N	2.84462600	1.43946100	-0.00264100
I	2.48592900	-1.58579900	0.00044900
C	4.30092700	1.37421600	-0.00698300
H	4.66891500	2.39616300	-0.00730500
H	4.63873300	0.84591500	0.88337600
H	4.63360400	0.84719500	-0.90005300
N	0.97211000	2.30357000	-0.00106700
C	-0.00292600	3.39164600	0.00560300
H	-0.65851200	3.27570700	-0.85534400
H	-0.58930000	3.33094500	0.92068400
H	0.53926100	4.33227700	-0.04419200
Cu	-1.22083700	0.24249300	0.00292300
I	-3.65046900	-0.36568800	-0.00108300

D

C	-0.68945400	0.63199900	-0.00004300
N	-0.00012900	2.73617400	0.00004200
N	-1.03851700	1.94633600	-0.00001800
I	-2.03188800	-0.94604300	0.00003500
C	-2.38641000	2.52520400	-0.00016500
H	-2.26742300	3.60506300	-0.00008000
H	-2.91035000	2.19406000	-0.89518900
H	-2.91056500	2.19398100	0.89470100
N	1.03839900	1.94648600	0.00003100
C	2.38620000	2.52556100	0.00012700
H	2.91020900	2.19441600	0.89510700
H	2.91035500	2.19445700	-0.89477500
H	2.26709800	3.60542500	0.00014400
C	0.68955400	0.63211600	0.00001400
I	2.03194600	-0.94602600	-0.00003300

3. Copies of ^1H and ^{13}C NMR Spectra

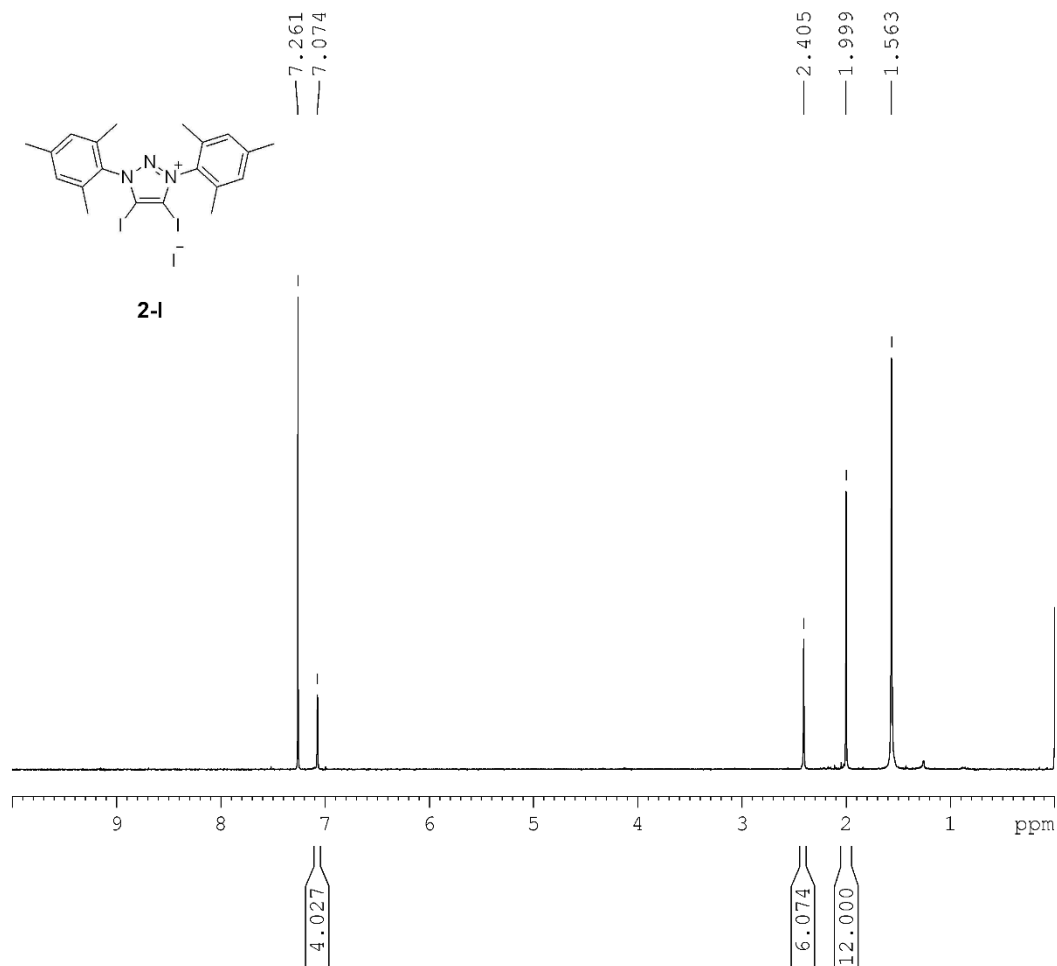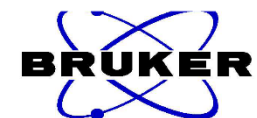

NAME xxy-4a-20161004
 EXPNO 1
 PROCNO 1
 Date_ 20161004
 Time_ 16.15
 INSTRUM spect
 PROBHD 5 mm PADUL 13C
 PULPROG zg30
 TD 32768
 SOLVENT CDCl3
 NS 8
 DS 0
 SWH 6393.862 Hz
 FIDRES 0.195125 Hz
 AQ 2.5625076 sec
 RG 575
 DW 78.200 usec
 DE 6.50 usec
 TE 295.0 K
 D1 1.00000000 sec
 TD0 1

===== CHANNEL f1 =====
 NUC1 1H
 P1 13.10 usec
 PL1 1.80 dB
 PL1W 8.92857742 W
 SFO1 400.1326008 MHz
 SI 32768
 SF 400.1300096 MHz
 WDW EM
 SSB 0
 LB 0.30 Hz
 GB 0
 PC 1.00

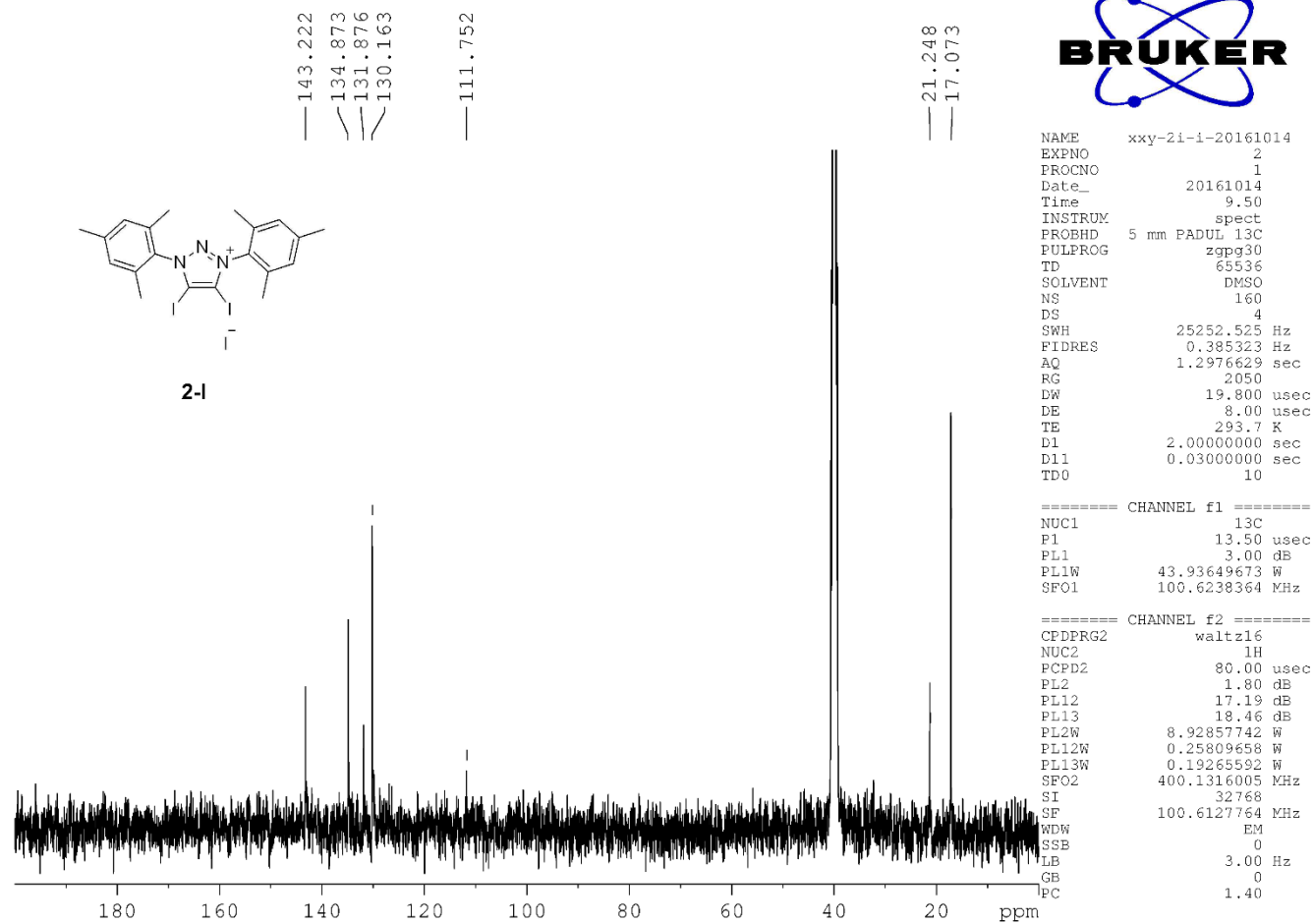

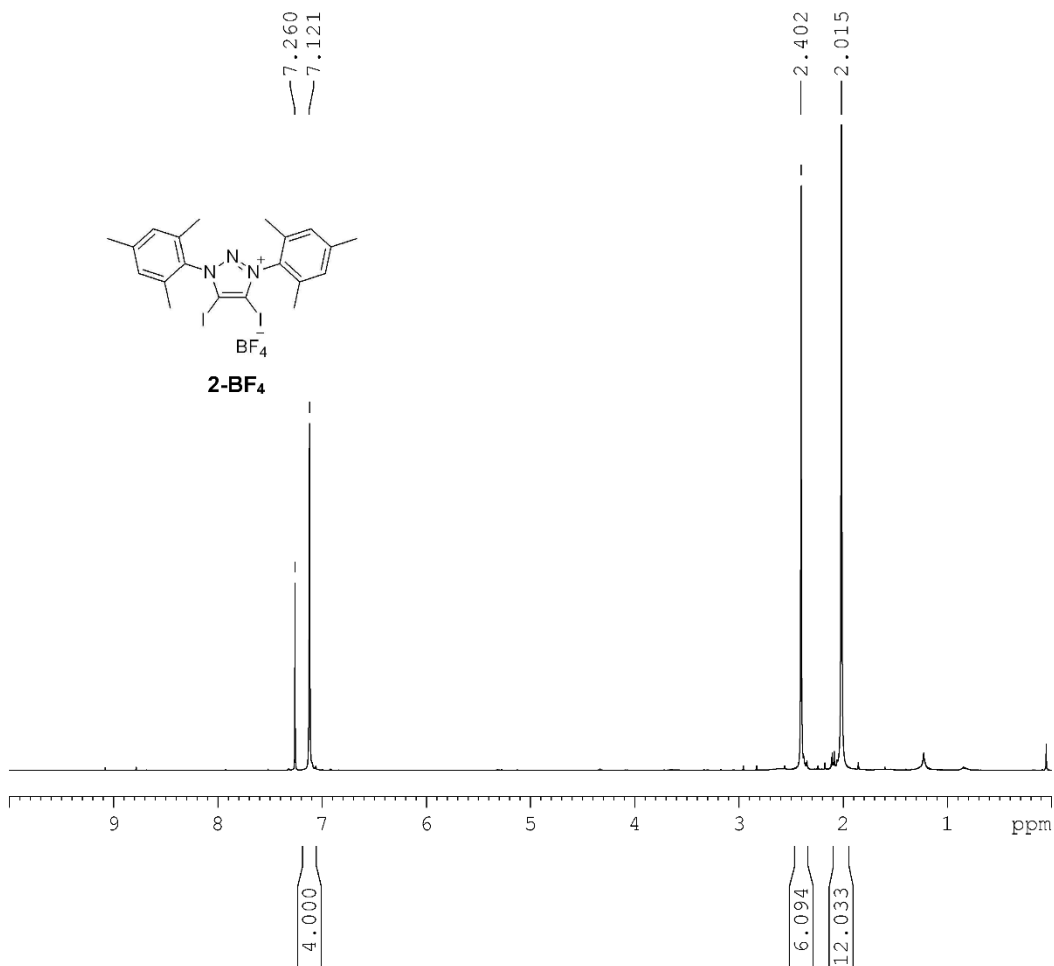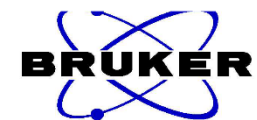

```

NAME      xxy-2i-agbf4-20161013
EXPNO     .
PROCNO    .
Date_     20161013
Time      17.09
INSTRUM   spect
PROBHD    5 mm PABUL 13C
PULPROG   zg30
TD        32768
SOLVENT   CDCl3
NS         8
DS         0
SWH        6393.862 Hz
FIDRES     0.195125 Hz
AQ         2.5625076 sec
RG         128
DW         78.200 usec
DE         6.50 usec
TE         293.2 K
D1         1.00000000 sec
TDC        .
  
```

```

===== CHANNEL f1 =====
NUC1       1H
P1         13.10 usec
PL1        1.80 dB
PL1W       8.92857742 W
SFO1       400.1326008 MHz
SI         32768
SF         400.1300096 MHz
WDW        EM
SSB        0
LB         0.30 Hz
GB         0
PC         1.00
  
```

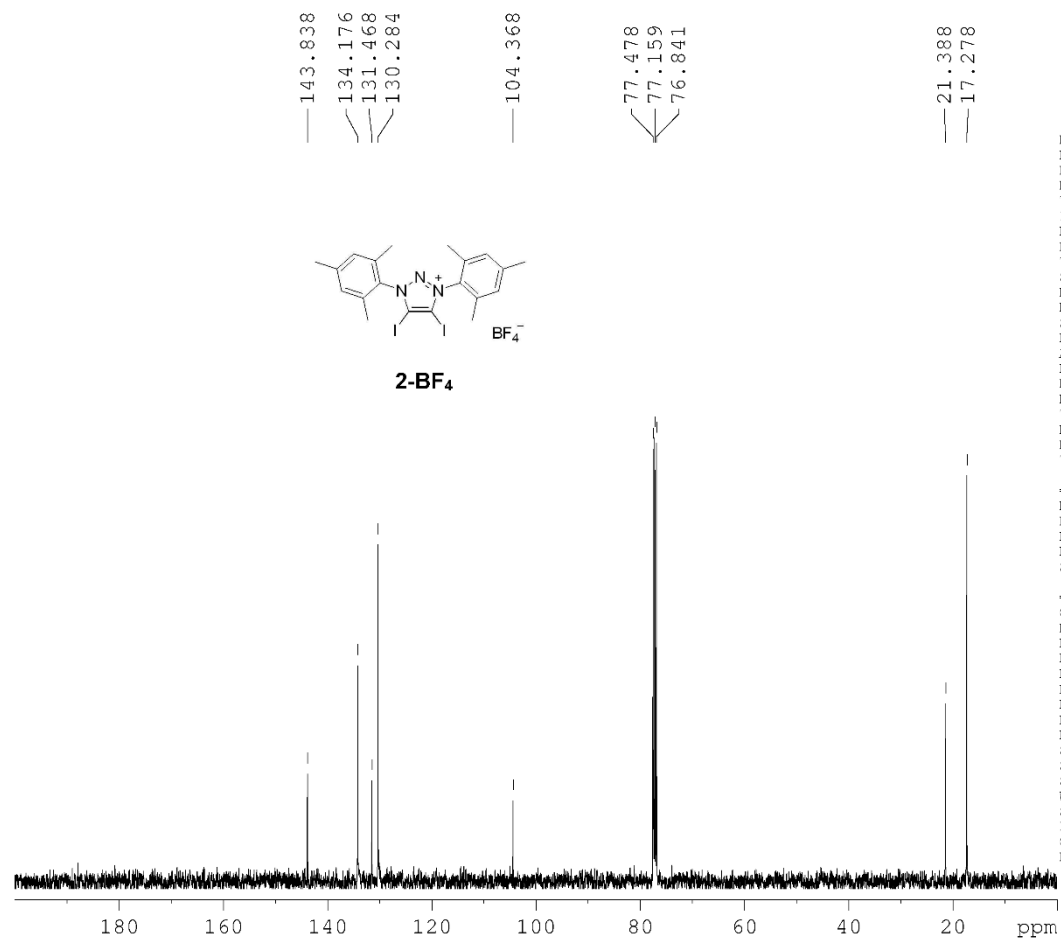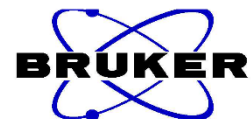

```

NAME      xxy-2i-agbf4-20161013
EXPNO     2
PROCNO    1
Date_     20161013
Time      17.14
INSTRUM   spect
PROBHD    5 mm PABUL 13C
PULPROG   zgpg30
TD         65536
SOLVENT   CDCl3
NS         24
DS         4
SWH        25252.525 Hz
FIDRES     0.385323 Hz
AQ         1.2976629 sec
RG         2050
DW         19.800 usec
DE         8.00 usec
TE         293.8 K
D1         2.0000000 sec
D11        0.0300000 sec
TD0        10

===== CHANNEL f1 =====
NUC1       13C
P1         13.50 usec
PL1        3.00 dB
PL1W       43.93649673 W
SFO1       100.6238364 MHz

===== CHANNEL f2 =====
CPDPRG2    waltz16
NUC2       1H
PCPD2      80.00 usec
PL2        1.80 dB
PL12       17.19 dB
PL13       18.46 dB
PL2W       8.92857742 W
PL12W      0.25809658 W
PL13W      0.19265592 W
SFO2       400.1316005 MHz
SI         32768
SF         100.6127681 MHz
WDW        EM
SSB        0
LB         3.00 Hz
GB         0
PC         1.40

```

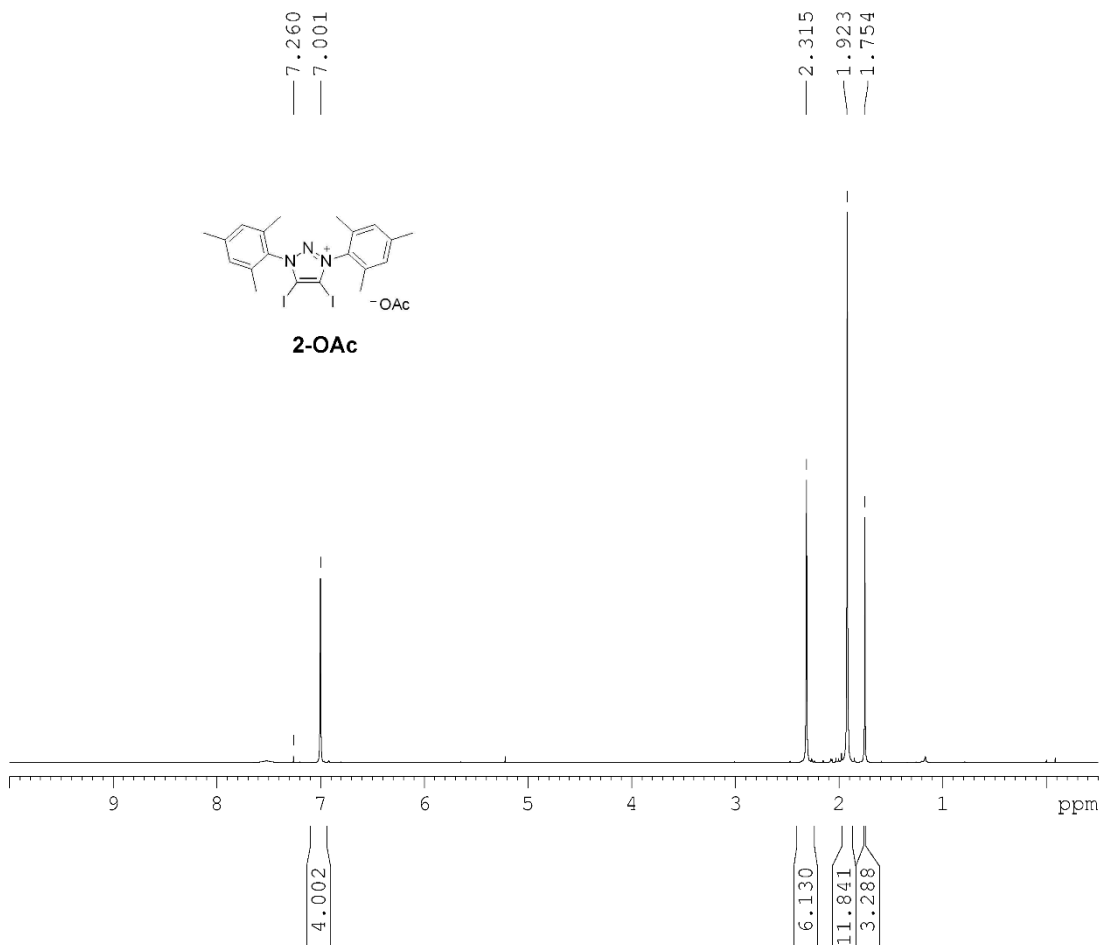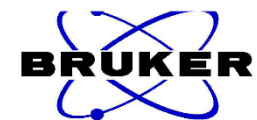

```

NAME      xxy-828ac-20180829
EXPNO     1
PROCNO    1
Date_     20180829
Time      14.07
INSTRUM   spect
PROBHD    5 mm PABBO BB-
PULPROG   zg30
TD        32768
SOLVENT   CDC13
NS         8
DS         0
SWH       6393.862 Hz
FIDRES    0.195125 Hz
AQ        2.5625076 sec
RG         64
DW        78.200 usec
DE         6.50 usec
TE        292.7 K
D1        1.00000000 sec
TD0       1
  
```

```

===== CHANNEL f1 =====
NUC1      1H
P1        10.40 usec
PL1       -1.00 dB
PL1W      17.01305389 W
SFO1      400.1326008 MHz
SI        32768
SF        400.1300119 MHz
WDW       EM
SSB       0
LB        0.30 Hz
GB        0
PC        1.00
  
```

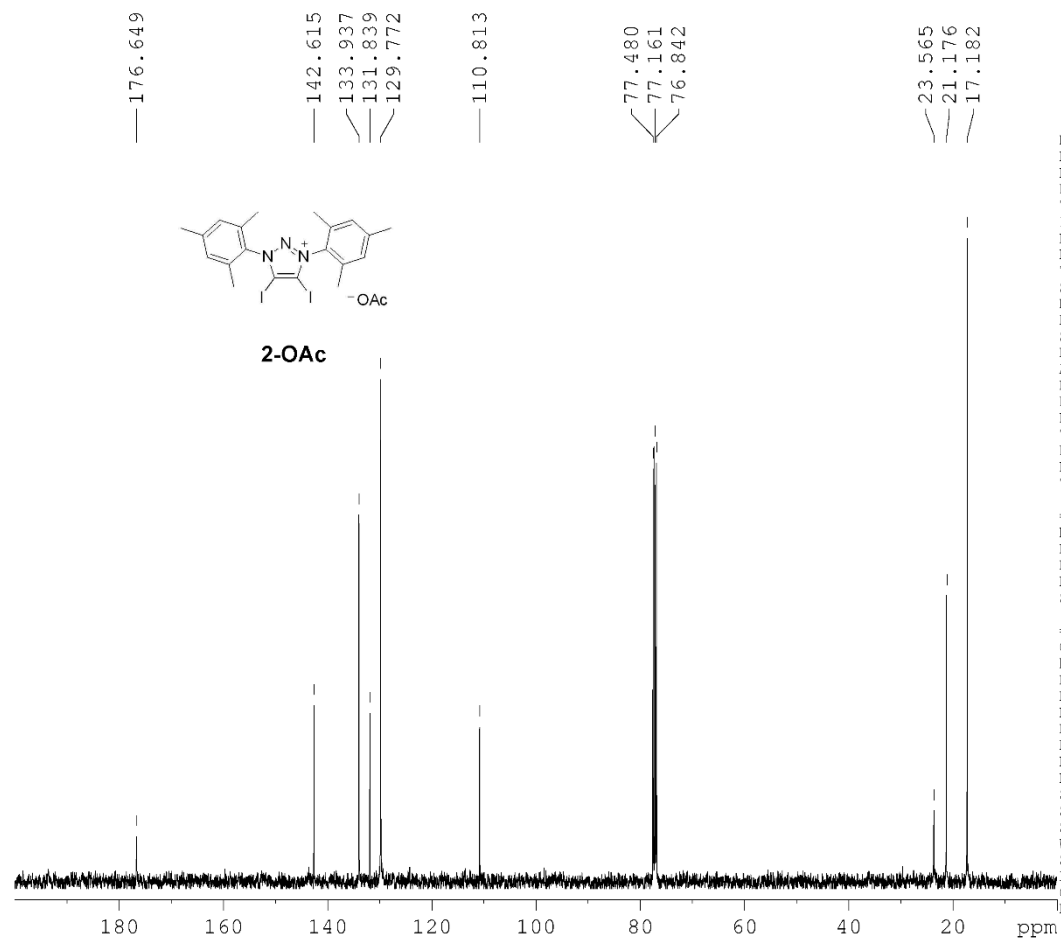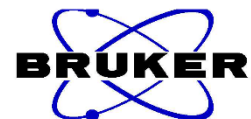

```

NAME      xxy-828ac-20180829
EXPNO     2
PROCNO    1
Date_     20180829
Time      14.12
INSTRUM   spect
PROBHD    5 mm PABBO BB-
PULPROG   zgpg30
TD        65536
SOLVENT   CDCl3
NS        32
DS        4
SWH       25252.525 Hz
FIDRES    0.385323 Hz
AQ        1.2976629 sec
RG        181
DW        19.800 usec
DE        6.50 usec
TE        293.4 K
D1        2.00000000 sec
D11       0.03000000 sec
TD0       3

```

```

===== CHANNEL f1 =====
NUC1      13C
P1        15.00 usec
PL1       2.00 dB
PL1W      55.31277084 W
SFO1      100.6238364 MHz

```

```

===== CHANNEL f2 =====
CPDPRG2   waltz16
NUC2      1H
PCPD2     80.00 usec
PL2       -1.00 dB
PL12      16.72 dB
PL13      15.50 dB
PL2W      17.01305389 W
PL12W     0.28759566 W
PL13W     0.38087484 W
SFO2      400.1316005 MHz
SI        32768
SF        100.6127797 MHz
WDW       EM
SSB       0
LB        3.00 Hz
GB        0
PC        1.40

```

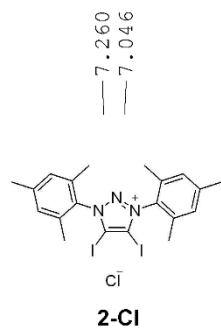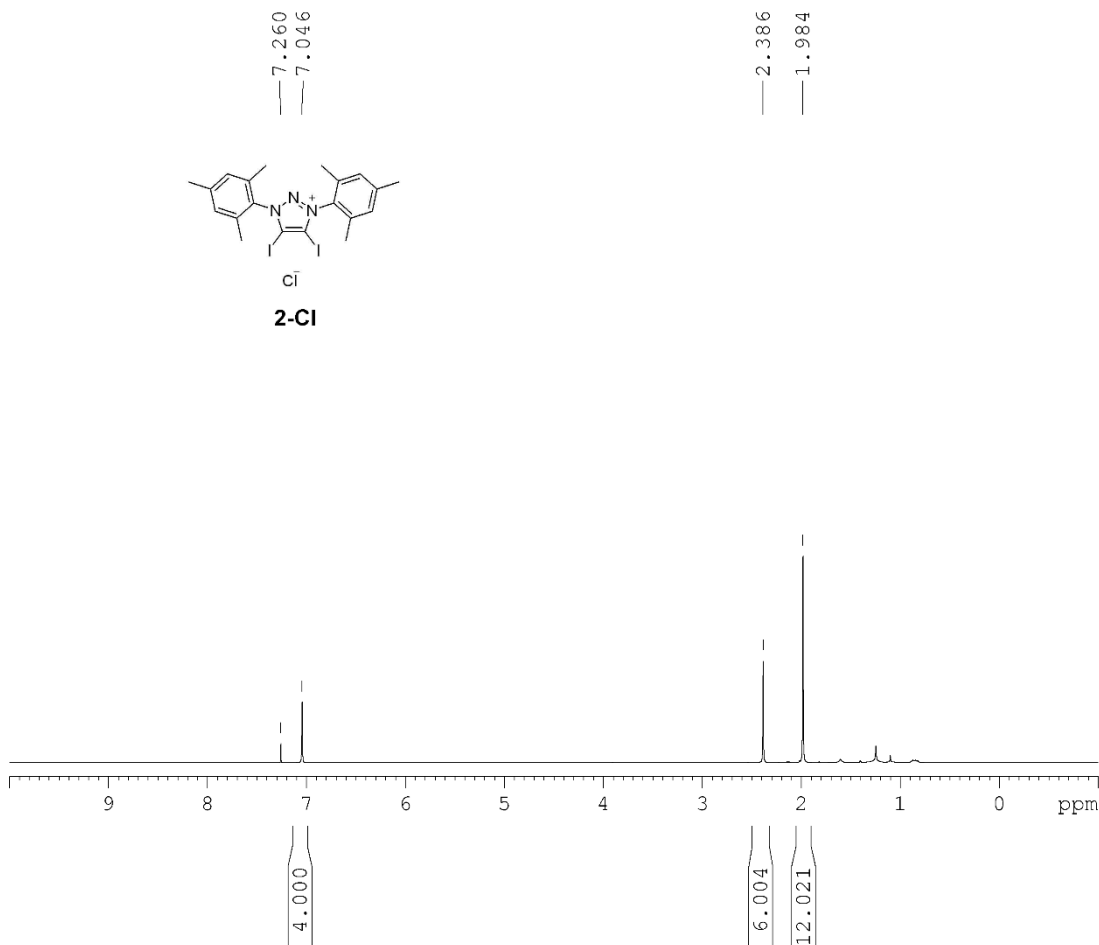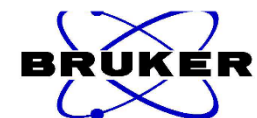

NAME xxy-10-05-2ic1
 EXPNO 1
 PROCNO 1
 Date_ 20191005
 Time_ 10.44
 INSTRUM spect
 PROBHD 5 mm PABBO BB-
 PULPROG zg30
 TD 32768
 SOLVENT CDCl3
 NS 8
 DS 0
 SWH 6393.862 Hz
 FIDRES 0.195125 Hz
 AQ 2.5625076 sec
 RG 322
 DW 78.200 usec
 DE 6.50 usec
 TE 295.4 K
 D1 1.00000000 sec
 TD0 1

===== CHANNEL f1 =====
 NUC1 1H
 P1 10.40 usec
 PL1 -1.00 dB
 PL1W 17.01305389 W
 SFO1 400.1326008 MHz
 SI 32768
 SF 400.1300095 MHz
 WDW EM
 SSB 0
 LB 0.30 Hz
 GB 0
 PC 1.00

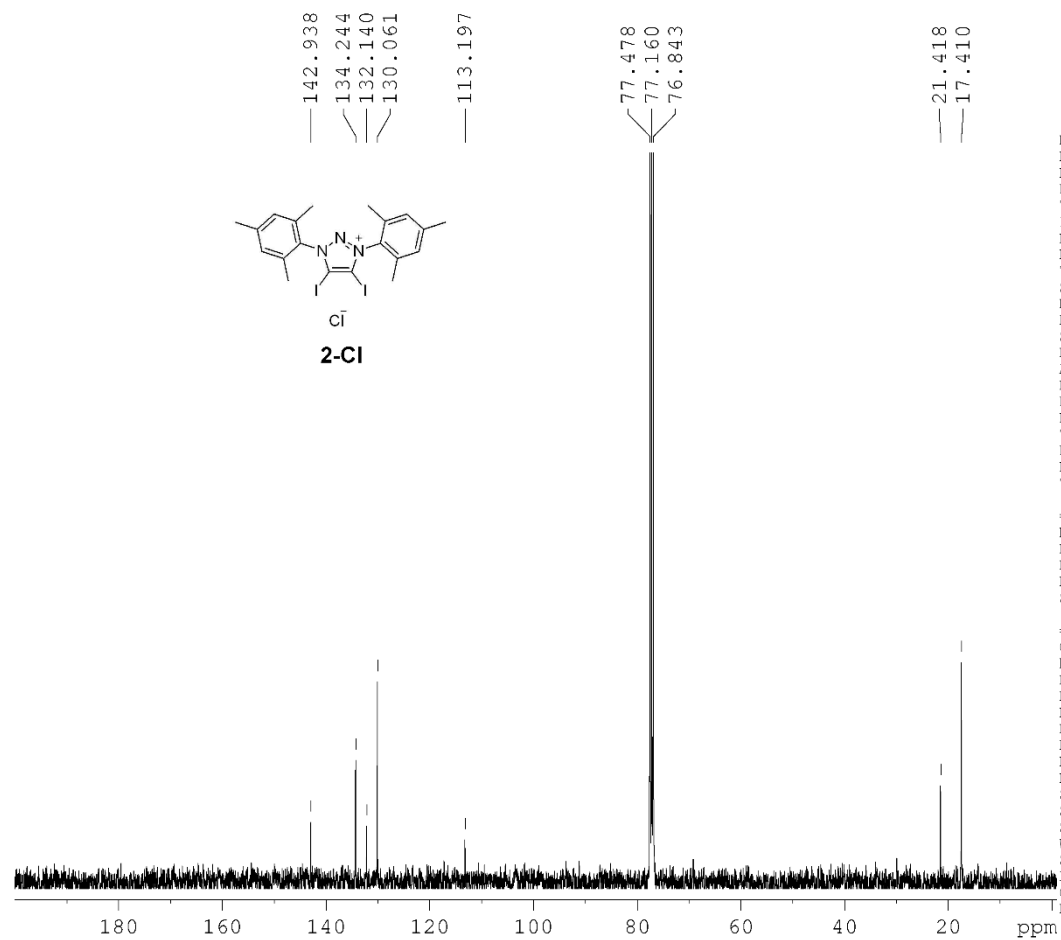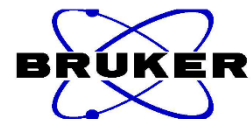

```

NAME      xxy-10-05-2ic1
EXPNO      2
PROCNO     1
Date_      20191005
Time       10.46
INSTRUM    spect
PROBHD     5 mm PABBO BB-
PULPROG    zgpg30
TD         65536
SOLVENT    CDCl3
NS         96
DS         4
SWH        25252.525 Hz
FIDRES     0.385323 Hz
AQ         1.2976629 sec
RG         2050
DW         19.800 usec
DE         6.50 usec
TE         296.0 K
D1         2.00000000 sec
D11        0.03000000 sec
TD0        3

```

```

===== CHANNEL f1 =====
NUC1       13C
P1         15.00 usec
PL1        2.00 dB
PL1W       55.31277084 W
SFO1       100.6238364 MHz

```

```

===== CHANNEL f2 =====
CPDPRG2    waltz16
NUC2       1H
PCPD2      80.00 usec
PL2        -1.00 dB
PL12       16.72 dB
PL13       15.50 dB
PL2W       17.01305389 W
PL12W      0.28759566 W
PL13W      0.38087484 W
SFO2       400.1316005 MHz
SI         32768
SF         100.6127556 MHz
WDW        EM
SSB        0
LB         3.00 Hz
GB         0
PC         1.40

```

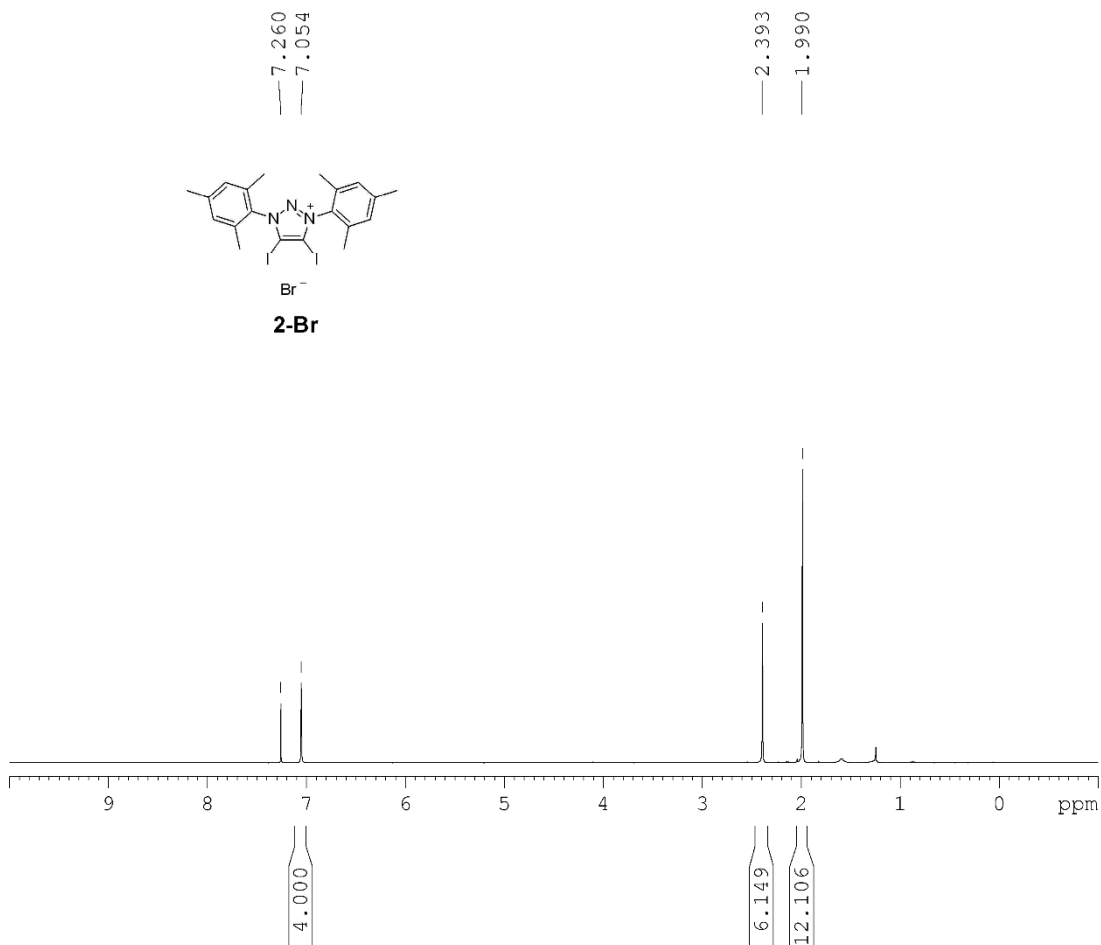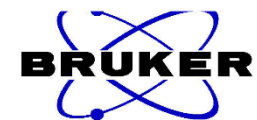

```

NAME      xxy-10-05-2ibr
EXPNO     1
PROCNO    1
Date_     20191005
Time      11.01
INSTRUM    spect
PROBHD     5 mm PABBO BB-
PULPROG    zg30
TD         32768
SOLVENT    CDCl3
NS         8
DS         0
SWH        6393.862 Hz
FIDRES     0.195125 Hz
AQ         2.5625076 sec
RG         322
DW         78.200 usec
DE         6.50 usec
TE         295.5 K
D1         1.00000000 sec
TD0        1

```

```

===== CHANNEL f1 =====
NUC1      1H
P1        10.40 usec
PL1       -1.00 dB
PL1W      17.01305389 W
SFO1      400.1326008 MHz
SI        32768
SF        400.1300095 MHz
WDW       EM
SSB       0
LB        0.30 Hz
GB        0
PC        1.00

```

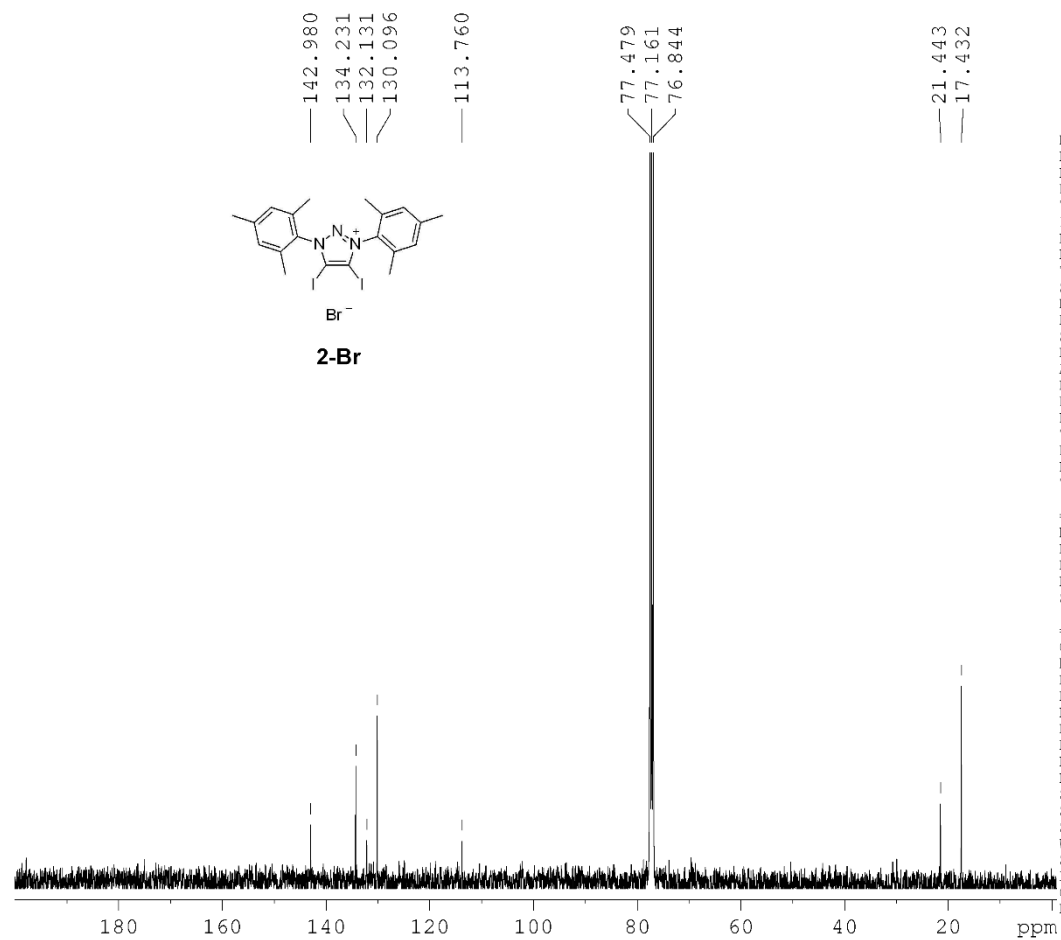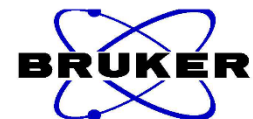

```

NAME      xxy-10-05-2ibr
EXPNO     2
PROCNO    1
Date_     20191005
Time      11.04
INSTRUM   spect
PROBHD    5 mm PABBO BB-
PULPROG   zgpg30
TD        65536
SOLVENT   CDCl3
NS        200
DS        4
SWH       25252.525 Hz
FIDRES    0.385323 Hz
AQ        1.2976629 sec
RG        2050
DW        19.800 usec
DE        6.50 usec
TE        296.3 K
D1        2.00000000 sec
D11       0.03000000 sec
TD0       3

```

```

===== CHANNEL f1 =====
NUC1      13C
P1        15.00 usec
PL1       2.00 dB
PL1W      55.31277084 W
SFO1      100.6238364 MHz

```

```

===== CHANNEL f2 =====
CPDPRG2   waltz16
NUC2      1H
PCPD2     80.00 usec
PL2       -1.00 dB
PL12      16.72 dB
PL13      15.50 dB
PL2W      17.01305389 W
PL12W     0.28759566 W
PL13W     0.38087484 W
SFO2      400.1316005 MHz
SI        32768
SF        100.6127550 MHz
WDW       EX
SSB       0
LB        3.00 Hz
GB        0
PC        1.40

```

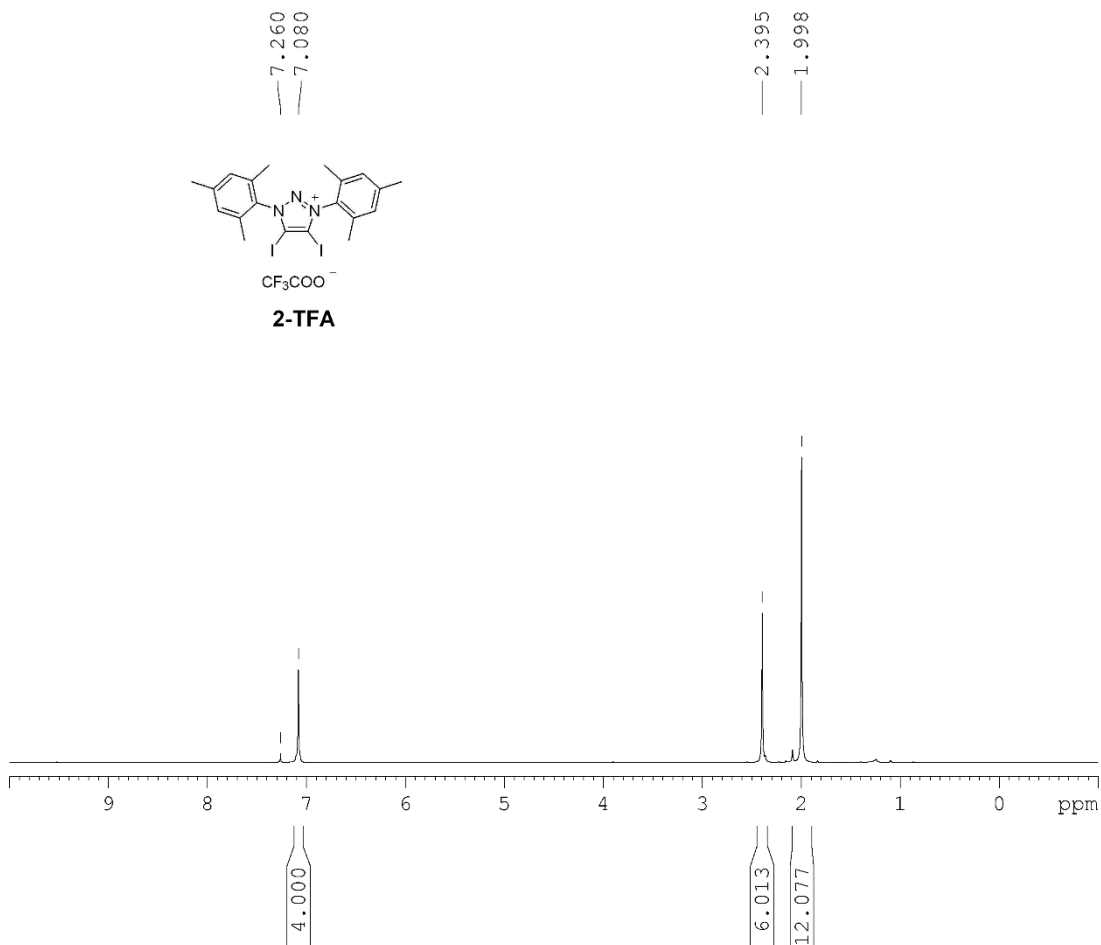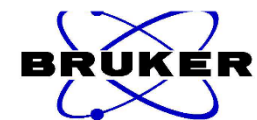

```

NAME      xxy-10-05-2itfa-2
EXPNO     1
PROCNO    1
Date_     20191005
Time      13.17
INSTRUM   spect
PROBHD    5 mm PABBO BB-
PULPROG   zg30
TD        32768
SOLVENT   CDCl3
NS         8
DS         0
SWH        6393.862 Hz
FIDRES     0.195125 Hz
AQ         2.5625076 sec
RG         181
DW         78.200 usec
DE         6.50 usec
TE         295.3 K
D1         1.00000000 sec
TD0        1
  
```

```

===== CHANNEL f1 =====
NUC1      1H
P1         10.40 usec
PL1        -1.00 dB
PL1W       17.01305389 W
SFO1      400.1326008 MHz
SI         32768
SF         400.1300096 MHz
WDW        EM
SSB         0
LB         0.30 Hz
GB          0
PC          1.00
  
```

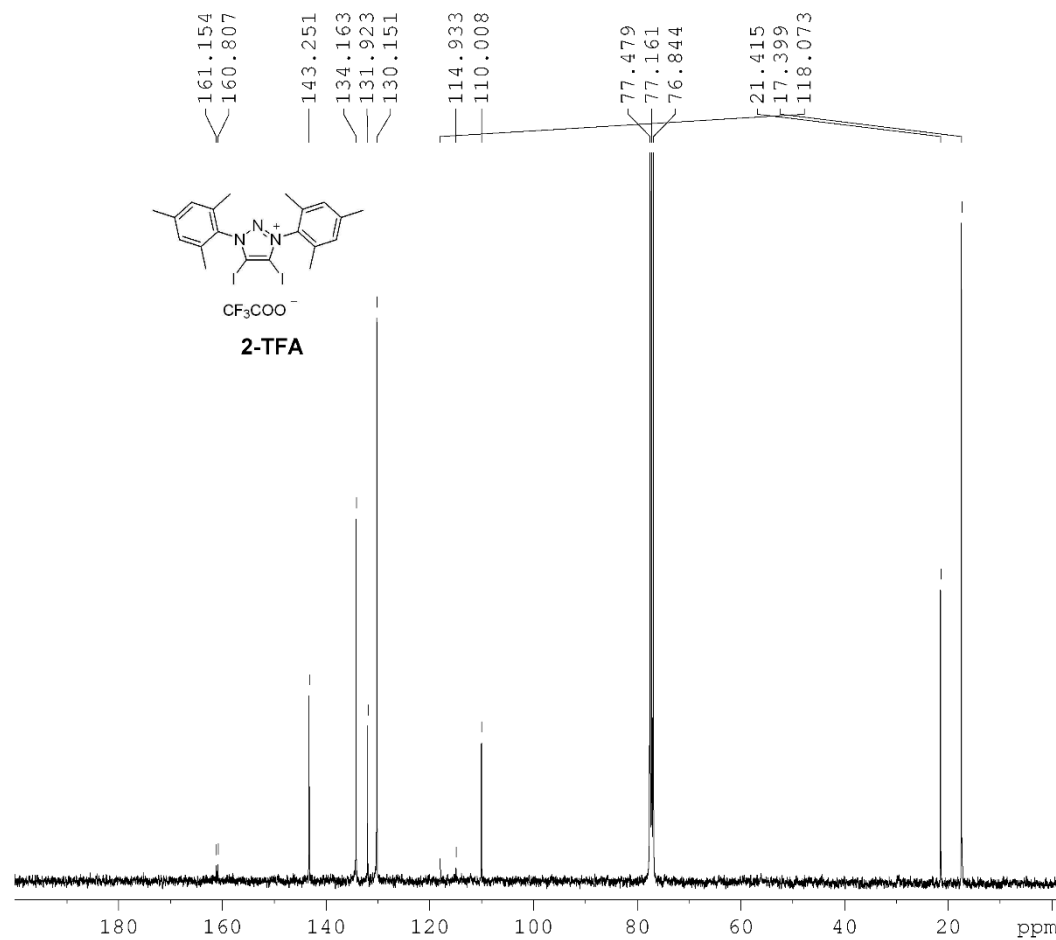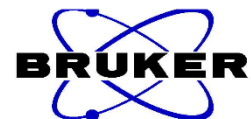

```

NAME      xxy-10-05-2itfa-2
EXPNO     2
PROCNO    1
Date_     20191005
Time      13.21
INSTRUM   spect
PROBHD    5 mm PABBO BB-
PULPROG   zgpg30
TD        65536
SOLVENT   CDCl3
NS        1032
DS        4
SWH       25252.525 Hz
FIDRES    0.385323 Hz
AQ        1.2976629 sec
RG        2050
DW        19.800 usec
DE        6.50 usec
TE        295.9 K
D1        2.00000000 sec
D11       0.03000000 sec
TD0       3

```

```

===== CHANNEL f1 =====
NUC1      13C
P1        15.00 usec
PL1       2.00 dB
PL1W      55.31277084 W
SFO1      100.6238364 MHz

```

```

===== CHANNEL f2 =====
CPDPRG2   waltz16
NUC2      1H
PCPD2     80.00 usec
PL2       -1.00 dB
PL12      16.72 dB
PL13      15.50 dB
PL2W      17.01305389 W
PL12W     0.28759566 W
PL13W     0.38087484 W
SFO2      400.1316005 MHz
SI        32768
SF        100.6127577 MHz
WDW       EM
SSB       0
LB        3.00 Hz
GB        0
PC        1.40

```

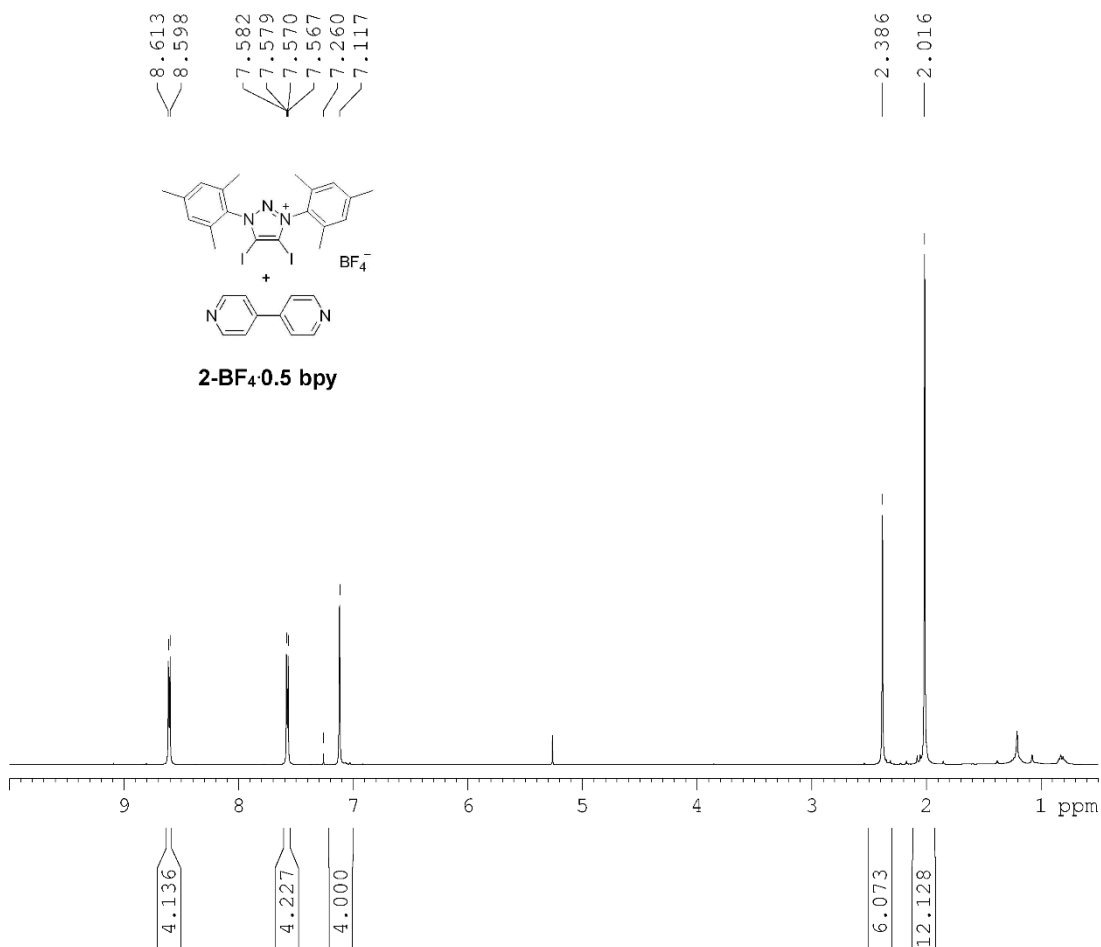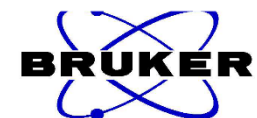

```

NAME      xxy-2ibpy-20180903
EXPNO     1
PROCNO    1
Date_     20180903
Time      15.58
INSTRUM   spect
PROBHD    5 mm PABBO BB-
PULPROG   zg30
TD        32768
SOLVENT   CDCl3
NS         8
DS         0
SWH       6393.862 Hz
FIDRES    0.195125 Hz
AQ        2.5625076 sec
RG         80.6
DW        78.200 usec
DE         6.50 usec
TE        292.6 K
D1        1.00000000 sec
TD0        1
  
```

```

===== CHANNEL f1 =====
NUC1      1H
P1        10.40 usec
PL1       -1.00 dB
PL1W      17.01305389 W
SFO1      400.1326008 MHz
SI        32768
SF        400.1300109 MHz
WDW       EM
SSB       0
LB        0.30 Hz
GB        0
PC        1.00
  
```

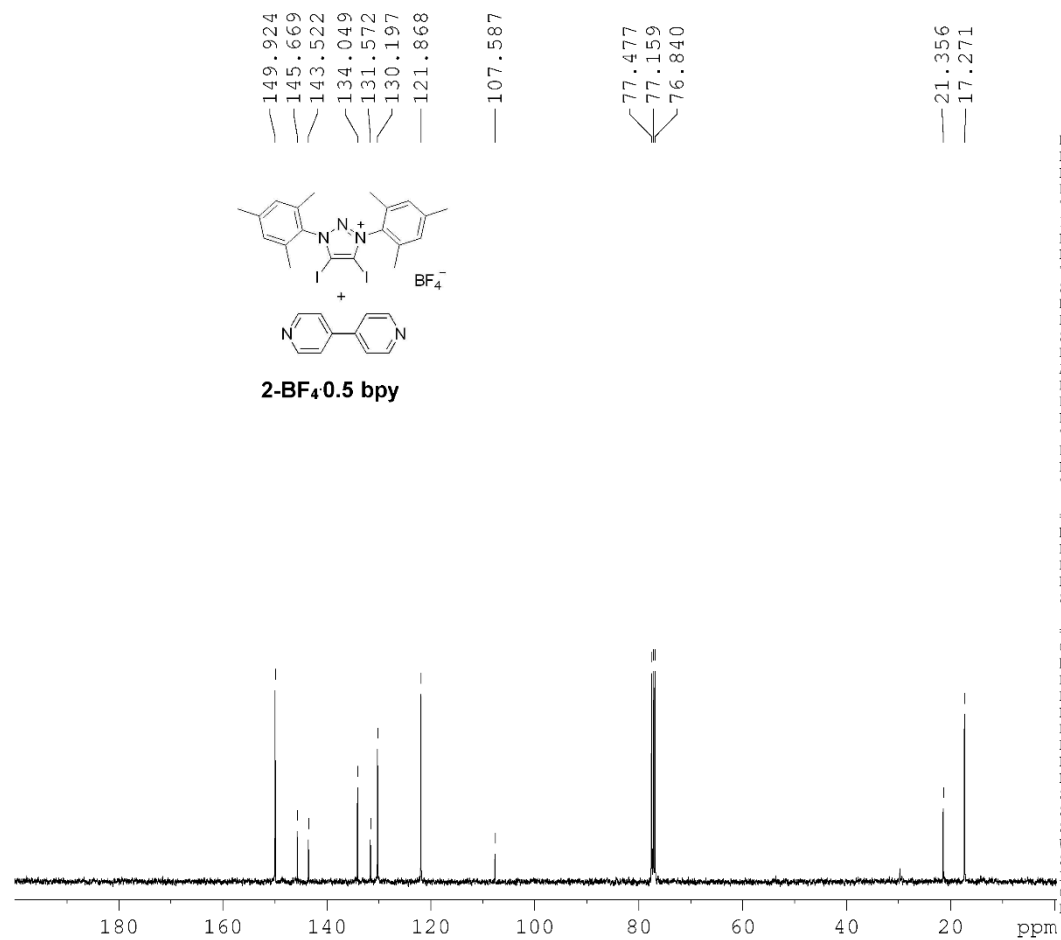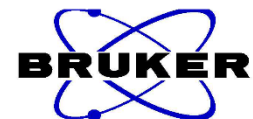

```

NAME      xxy-2ibpy-20180903
EXPNO     2
PROCNO    1
Date_     20180903
Time      15.48
INSTRUM   spect
PROBHD    5 mm PABBO BB-
PULPROG   zgpg30
TD        65536
SOLVENT   CDCl3
NS        64
DS        4
SWH       25252.525 Hz
FIDRES    0.385323 Hz
AQ        1.2976629 sec
RG        181
DW        19.800 usec
DE        6.50 usec
TE        293.5 K
D1        2.00000000 sec
D11       0.03000000 sec
TD0       3

```

```

===== CHANNEL f1 =====
NUC1      13C
P1        15.00 usec
PL1       2.00 dB
PL1W      55.31277084 W
SFO1      100.6238364 MHz

```

```

===== CHANNEL f2 =====
CPDPRG2   waltz16
NUC2      1H
PCPD2     80.00 usec
PL2       -1.00 dB
PL12      16.72 dB
PL13      15.50 dB
PL2W      17.01305389 W
PL12W     0.28759566 W
PL13W     0.38087484 W
SFO2      400.1316005 MHz
SI        32768
SF        100.6127711 MHz
WDW       EM
SSB       0
LB        3.00 Hz
GB        0
PC        1.40

```
